# Double-Blind, Single-Center, Randomized Three-Way Crossover Trial of Fitted, Thin, and Standard Condoms for Vaginal and Anal Sex: C-PLEASURE Study Protocol and Baseline Data

**DOI:** 10.2196/12205

**Published:** 2019-04-23

**Authors:** Aaron J Siegler, Elizabeth M Rosenthal, Patrick S Sullivan, Lauren Ahlschlager, Colleen F Kelley, C Christina Mehta, Reneé H Moore, Eli S Rosenberg, Michael P Cecil

**Affiliations:** 1 Rollins School of Public Health Department of Behavioral Sciences and Health Educaiton Emory University Atlanta, GA United States; 2 University at Albany School of Public Health Department of Epidemiology State University of New York Albany, NY United States; 3 Department of Epidemiology Rollins School of Public Health Emory University Atlanta, GA United States; 4 School of Medicine Emory University Atlanta, GA United States; 5 Rollins School of Public Health Department of Biostatistics and Bioinformatics Emory University Atlanta, GA United States; 6 TheyFit, LLC Covington, GA United States

**Keywords:** condoms, HIV prevention, sexual health, clinical trial

## Abstract

**Background:**

Male condoms are underused despite their ability to prevent transmission of HIV and other sexually transmitted infections. The perception of decreased sexual pleasure and poor condom fit are major contributors to condom nonuse.

**Objective:**

The purpose of this study was to compare event-level performance and pleasure using fitted, thin, and standard condoms among men who have sex with men (MSM) and men who have sex with women (MSW). We also sought to assess condom type preference. We present the study design and enrollment data from the trial.

**Methods:**

This study recruited sexually active men aged 18 to 54 years in Atlanta, Georgia, United States. We enrolled 252 MSM and 252 MSW in a double-blind, 3-way randomized crossover trial with conditions of fitted, thin, and standard condoms. A permuted block randomization scheme was used to assign each participant to the sequence in which they received each type of study condom. After a baseline screening and enrollment visit, randomized participants were followed for at least 6 and up to 12 weeks depending on their use of study condoms in each 2-week period between scheduled, in-person study visits. Participants were instructed to complete mobile-optimized coital logs as soon as possible after using condoms for anal or vaginal sex acts. The logs collected event-level pleasure and performance measures for the study condoms as well as other relevant data. A questionnaire was administered at the final study visit to assess overall study condom preference.

**Results:**

The study enrolled 252 MSM and 252 MSW, a total of 504 participants. MSM and MSW study arms were similar for a number of key traits including race and ethnicity, marital status, self-rated condom experience, and recent experience of condom failure. Men in the MSM arm were older, however, and fewer MSM were students. The majority of participants in both arms rated themselves as very experienced with using condoms, and the majority had used condoms recently. Over one-third of participants in each arm reported experiencing condom failure in the last 6 months.

**Conclusions:**

This is the first condom trial to compare the performance of standard, thin, and fitted condoms and to use pleasure and preference as primary outcomes. Given the disparate impact of HIV on MSM, equal enrollment of MSM and MSW was a key feature of this study. Trial results may inform an FDA label indication for anal sex and provide new information regarding the relative performance of different types of condoms.

**Trial Registration:**

ClinicalTrials.gov NCT02753842; https://clinicaltrials.gov/ct2/show/NCT02753842 (Archived by WebCite at http://www.webcitation.org/76RLTFyf0)

**International Registered Report Identifier (IRRID):**

DERR1-10.2196/12205

## Introduction

Male condoms effectively prevent HIV and sexually transmitted disease (STD) transmission but are underused in large part due to perceived reductions in the experience of pleasure. In the United States, diagnosis of chlamydia, gonorrhea, and syphilis has increased in each of the last 4 years, with approximately 2.3 million diagnoses in 2017 [1]. HIV incidence remains high, with an estimated 38,500 incident infections in 2015 [2]. Condoms have high efficacy in preventing HIV transmission [3], and the highest priority for any condom promotion effort is to change factors that lead to condom nonuse [4].

A broad array of factors influence condom use spanning policy, cultural, interpersonal, and individual levels. Condom accessibility through public supply chains, which are largely determined by purchase and distribution policies, has been demonstrated to impact condom uptake [5,6]. Cultural norms may also influence condom use, both when values are held that discourage condom use [7] and when values are held that make condom use normative [8]. Relationship-level variables may be important, including marital status and trust in a particular relationship [9,10]. Individual-level skills also matter, in particular condom use self-efficacy and the ability to negotiate condom use in a relationship [11], which in turn may be inextricably linked to cultural roles and norms [12,13]. Even if nearly ideal conditions were reached (eg, readily available condoms for users with self-efficacy amid supportive community norms) condoms might remain underused due to the widely held belief and perception that condoms decrease sexual pleasure [14-17].

Across several studies, between one-third and one-half of condom users report poor condom fit [14,18-20]. Men who have sex with men (MSM) who either perceive condoms as too tight [21] or report larger than average penis size [18] were more likely to report unprotected sex. One reason for this is that men who perceive poor condom fit have been found to be more likely to report reduced pleasure due to condom use [14,20].

The premise of fitted condoms is that better fitting condoms may enhance perceptions of pleasure or influence overall preference for men considering condom use. There are two biologically plausible hypotheses for this premise. First, men reporting larger penile size are more likely to describe standard condoms as feeling tight [19,21], and this tightness could lead to decreased perceptions of pleasure. Second, men who report smaller penis size are more likely to describe condoms as feeling loose [19], and this additional slack (circumference) or rolled (length) latex could lead to decreased perceptions of pleasure.

MSM are a group meriting particular consideration because they are disproportionately impacted by HIV, accounting for 2 out of every 3 new HIV diagnoses in the United States, with anal sex being the predominant mode of transmission for this group [22]. Previous estimates of clinical condom failure (slippage or breakage) during anal sex often have not measured failure at the event level or used prospective designs. The two studies that assessed clinical condom failure prospectively at the event level reported failure in 6.3% [23] and 6.9% [24] of anal sex acts.

The purpose of this research was to better understand whether different types of condoms lead to different experiences of pleasure and clinical failure. This trial compared the performance of fitted, thin, and standard condoms.

## Methods

### Study Design and Aims

**Figure 1:**
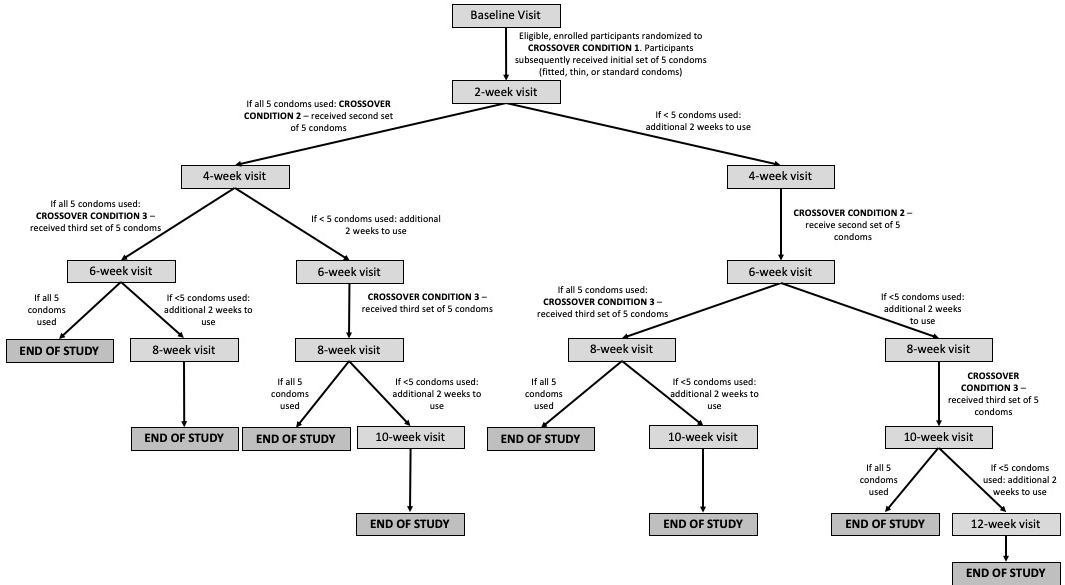
Study visit flowchart.

This study was a double-blinded, randomized crossover trial of 252 MSM and 252 men who have sex with women (MSW). Participants were enrolled from May 2016 to May 2017. Over a series of in-person study visits, participants received in randomized order a set of 5 fitted condoms, a set of 5 thin condoms, and a set of 5 standard condoms. Participants were followed for 6 to 12 weeks, depending on their use of study condoms in each 2-week period between study visits (Figure 1). Event-level data based on a home coital log were collected regarding pleasure and total clinical failure, and data regarding overall condom preference were collected at the final study visit.

As specified at trial registration [25], we conducted this trial with the objectives of establishing label indications for pleasure and patient preference for fitted condoms (aims 1 and 2), establishing a label indication for anal sex for fitted, thin, and standard condoms (aim 3), and establishing a label indication for decreased clinical failure of fitted condoms for anal sex (aim 4). This will be accomplished by comparing fitted condoms with standard condoms regarding levels of reported pleasure as determined by rating per condom use event (aim 1), comparing fitted condoms with standard condoms regarding preference as determined by ranking of the two conditions at the study conclusion (aim 2), assessing the total clinical failure rate of each type of condom (fitted, thin, standard) for anal sex among MSM relative to a cut-point to be determined by the US Food and Drug Administration (FDA) (aim 3), and comparing fitted condoms with standard condoms regarding total clinical failure for anal sex among MSM (aim 4). Detail around the hypotheses and rationale for each primary aim as well as secondary aims of the study and other areas of interest are provided in Multimedia Appendix 1.

### Ethics

The study was conducted in accordance with Title 21 US Code of Federal Regulations (CFR) Part 11 and Good Clinical Practice guidelines. The researchers obtained Emory University institutional review board (IRB) approval for the protocol and informed consent forms prior to initiating the study. All participants signed consent forms. All changes to the protocol were submitted to the Emory University IRB for review and approval as appropriate. The principal investigator followed the requirements of the Emory University IRB on periodic reporting of the progress of the study, reporting of serious or unexpected adverse events, and safety monitoring reports. Participants were informed that collected data were intended for publication and that individual details would be de-identified and stored in a secure, password-protected location available only to members of the research team. Additional ethical details can be found in Multimedia Appendix 2.

### Study Population and Recruitment

Primary recruitment for both MSM and MSW was face-to-face venue-based recruitment that took place in a variety of public and private venues in Atlanta where men congregate. Study staff also used secondary recruitment methods that included flyers, paid online advertisements, and recruitment from previous studies. Study sites were the Rollins School of Public Health at Emory University and the Emory Programs, Research, and Innovation in Sexual Minority Health research site. Eligible participants were HIV-negative at their baseline test, aged 18 to 54 years, lived in the Atlanta metro area, and were currently sexually active. We enrolled 252 HIV-negative MSM and 252 HIV-negative MSW. For purposes of study assignment, men were eligible for the MSM arm if they intended to only have sex with other men in the next 12 weeks, and men were eligible for the MSW arm if they intended to only have sex with women in the next 12 weeks. Per FDA guidance [26], only individuals who were willing to be the insertive partner for use of study condoms and therefore best able to ascertain study outcomes were eligible. Further details around participant inclusion criteria as well as recruitment procedures are provided in Multimedia Appendix 1.

Eligibility was assessed in three stages: (1) recruitment screening of less sensitive criteria such as age, (2) phone screening of more sensitive criteria such as genital piercing, and (3) in-person (baseline) screening for reassessment of all eligibility criteria in addition to a negative point-of-care HIV test. Prior to the baseline visit, participants determined their fitted condom size after being mailed instructions and a fitting tool consisting of a paper template graduated with nonsequential numbering and lettering. Multimedia Appendix 1 details participant retention procedures.

**Figure 2:**
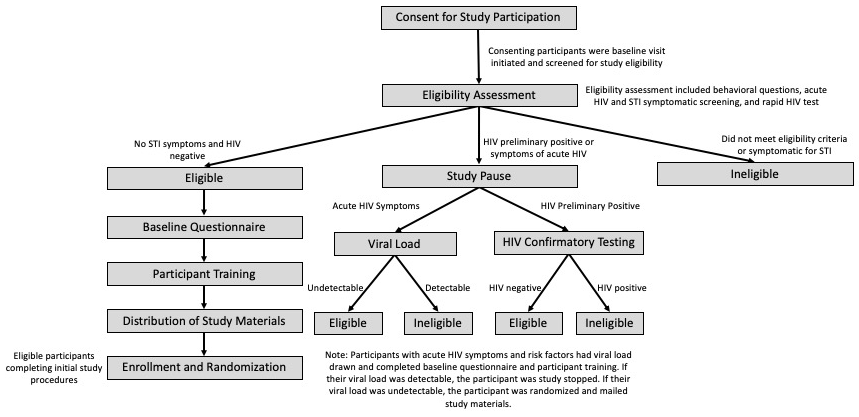
Baseline visit structure.

### Study Product

For purposes of this study, a standard condom is defined by dimensions commonly sourced by the United Nations Population Fund: 185 mm (±10 mm) in length, 53 mm (±2 mm) in width, and 70 microns (±10 microns) in thickness (Lai Peng Lim, BS, email communication, June 23, 2015). Thin condoms for this study were of identical width and length to standard but 50 microns (±5 microns) thick. Fitted condoms with a thickness of 70 microns (±10 microns) were produced in a range of sizes. Participants were given two 10-mL packets per condom of commercially available, condom-compatible water-based lubricants in plain foil packets. All condoms and lubricant used in the study were manufactured for the study by Karex Berhad. See Multimedia Appendix 1 for more information regarding study product.

### Randomization, Crossover, and Blinding Procedures

Within each study arm (MSM or MSW), the crossover condition order (the order in which condoms were provided) was determined by permuted block randomization as indicated by the clinical data management system (CDMS). Eligible study participants were randomized to 1 of 6 orders, with the 6 orders balancing the allocation of conditions (Multimedia Appendix 2). Each participant was given 5 of each study condom set and had up to 4 weeks to use all 5 condoms. If all 5 condoms in a set were used by the end of the 2-week follow-up period, participants were crossed over to the next condition. If not, participants were given an additional 2-week period to use condoms in that condition. The purpose of this structure was to maintain realistic use periods for study condoms; national survey data indicate that many men in the age range included in this study report sex at a rate that equates to between 1 and 2 sex acts per week [27,28].

In this closed label trial, study condoms were manufactured in plain foil packaging with identifying 2-digit random codes printed on each foil. Blinding of study staff was role-based; the study statistician and the principal investigator, who are responsible for analyses and reporting results to the FDA, will be blinded until after the initial analysis of study results has been conducted. To allow for blinded participants to identify preferred condoms (aim 2), we provided condom sets in color-coded bags. We selected colors that could accommodate common forms of color vision deficiency. Further information on study blinding is available in Multimedia Appendix 1.

### Study Visit Procedures

#### Enrollment/Baseline Visit

A visual depiction of the baseline visit structure is provided in Figure 2. Upon arrival at the visit, all participants completed informed consent and eligibility assessment. Participants received HIV counseling, rapid testing, and test results per US Centers for Disease Control and Prevention guidelines [29]. Participants with confirmed HIV infection, detectable viral loads, or with self-reported STD or symptoms for STD were ineligible to continue the study. We referred these participants to appropriate care (Multimedia Appendix 2). If the participant self-reported symptoms of acute HIV infection and an exposure risk in the past 30 days, blood was drawn for HIV viral load testing with results usually returned within two days. Those with detectable viral loads were study stopped and referred to care, with test results shared with providers upon participant consent; those with undetectable viral loads continued in the study.

Eligible participants completed a self-administered electronic survey and were trained on using the home coital log and correct use of condoms (Multimedia Appendix 2). Participants were instructed to only use study lubricant when using condoms. Guidance from the World Health Organization found substantial evidence that use of additional water-based lubricant decreases anal sex failure and that lubricant use is normative among many MSM [30]. Therefore, MSM were instructed to use study lubricant for all anal sex acts. The same guidance found equivocal evidence for MSW, with some studies showing a small benefit of additional lubricant and a similar number showing no benefit. Given limited evidence of benefit, and MSW not normatively using lubricant during condom-protected sex [31], we instructed MSW to use lubricant as needed or desired. Participants were scheduled for follow-up visits and provided study lubricant, printed study materials, and a biohazard bag to return any broken condoms.

#### Follow-Up Visits

A visual depiction of follow-up visit events is provided in Multimedia Appendix 3. At each follow-up visit, study staff performed a manual count of returned condoms. Staff compared the number of returned unused or broken condoms to the number of condom uses and breakages reported in home coital logs during the study period. If there were discrepancies, study staff worked with the participant to resolve them. If participants reported losing condoms at interim study visits, we replaced their lost stock. We conducted adverse event screening for potential partner pregnancy, self-reported acute HIV symptoms, self-reported STD or symptoms for STD, instances of condom failure, and side effects from condom or lubricant use (based on determination by the independent study clinician). Study participants screening positive for these events were referred to care (Multimedia Appendix 2). All assessments at follow-up visits were recorded on electronic case report forms (eCRFs). Study staff instructed participants to throw away any unused study condoms from the previous study period.

#### Coital Log Description

For event-level measurement, we used a mobile-optimized, Web-based home daily coital log that was 21 CFR Part 11–compliant. We anticipated that use of this system would minimize recall bias due to shorter time period for recall [32]; moreover, previous sexual health research has found that Web diaries produce improved data relative to other methods with longer recall periods [33]. Electronic diaries have an additional benefit over paper-based recall systems in the form of time stamping to accurately determine the time of form completion, and they allow for question piping/logic that converts otherwise complex paper forms into a sequence of brief, easily intelligible questions. Participants were instructed to complete coital logs as soon as possible following any vaginal or anal sex acts. Further, participants received an autogenerated coital log reminder either via text or email every 24 hours to check-in and catch any unreported sex events. The first coital log question determined whether a participant had sex since their last coital log entry. If no was selected, the coital log entry was complete. If yes, the coital log proceeded to query the sexual event. Participants not completing the coital log regularly were contacted by study staff to encourage more consistent completion. To further incentivize regular use of the coital log and therefore minimize recall bias, participants who completed at least 10 coital logs during each 2-week study period were compensated an extra $15 at their next study follow-up visit. Participants were informed that incentives were provided to encourage daily interaction with the system; equal compensation was provided for completions that reported sex and completions that reported no sex. For participants without access to Web browsers, we provided mobile phones.

### Measures

#### Electronic Case Report Forms

Study staff collected key data using eCRFs. For the baseline visit, information collected included eligibility criteria, basic demographic information, and acute HIV and STD symptoms. For follow-up visits, eCRFs included information regarding documentation of condition crossover, number of condoms distributed and returned, adverse events, and study stops.

#### Baseline Survey

The baseline survey included questions in the domains of (1) sexual history, (2) condom history, (3) sexual dysfunction, (4) condom slippage and breakage, (5) lubricant use, (6) therapeutic methods, (7) condom fit and feel, (8) condom perceptions, (9) self-efficacy around condom use, (10) HIV and STD history, (11) partner history, and (12) pleasure at last sex. The baseline questionnaire, which annotates the question source for each area of assessment, has been provided in Multimedia Appendix 4. Average completion time was 30 to 45 minutes.

#### Coital Log Measures

The coital log assessed the outcome of pleasure (aim 1) and the outcomes of clinical failure (aims 3 and 4). Based on a literature search and consultation with experts, we identified no extant event-level scale to assess aim 1. Therefore, we developed and validated the Event-level Male Sexual Pleasure Scale (EMSexPleasure), described elsewhere [34]. International Organization for Standardization (ISO) guidance defines clinical failure as combined clinical breakage and slippage [35], and we will follow ISO guidance reporting clinical failure for aims 3 and 4. For instance, any condom failure in which breakage and slippage occur for the same condom will be counted as a single failure for calculation of total clinical failure. The coital log also measured event-level domains regarding the nature and context of condom use: (1) date and time of report (source: ISO); (2) whether a study condom, other condom, or no condom was used (ISO); (3) partner name (cohort study of MSM [36]); (4) lubricant use (ISO); (5) type of sex act (ISO); and (6) drug or alcohol use by participant (cohort study of MSM).

We developed a procedure in the coital log to minimize error in self-report of clinical failure events. After initial completion of questions, the system autogenerated a message that provided a summary of participant’s self-report data, with an option to confirm it or to correct it. This message was provided for all reports, whether clinical failure was reported or not reported. For example, for a participant reporting that a condom broke but did not slip, the participant was asked, “You told us that this condom broke but did not slip. Is this correct?” Response options were yes, which led to continuation of the coital log, and no, which led to reinitiation of questions regarding condom failure.

To prevent recall bias from unduly influencing data, we established a set of rules regarding coital log completion at study events. At a study event, if a participant reported using study condoms but had not completed coital logs for them, we allowed a maximum of the past two condom uses to be reported. In these reports, participants entered data regarding clinical failure outcomes (aims 3 and 4) but not regarding event-level pleasure (aim 1) due to the higher potential recall bias for pleasure, which was considered an ephemeral phenomenon.

#### Follow-Up Visit Measures

At crossover visits, participants completed a self-administered behavioral survey in the domains of (1) perceived condom fit of the last study condom used, (2) perceived crossover condition, (3) new sexual partners, and (4) condom preference (at applicable visits). If participants reported any condom use that had not been previously recorded with coital logs, they were allowed to enter coital log data for up to their two most recent condom uses.

#### End Line Measures

Preference, the outcome measure for aim 2, was measured at the final study visit. For each of 3 possible combinations of 2 crossover conditions (standard/thin, thin/fitted, standard/fitted), there was a paired comparison asking participants to select their preferred condom between the 2 relevant study conditions. To maintain blinding, preference question response options were the color assigned to each condom type.

### Statistical Considerations

#### Statistical Power and Sample Size

For aim 2 at 80% and 90% power, assuming 80% retention, the minimum underlying values of fitted-condom preference π̂_2,1_ that would be detectable as significantly higher than 0.5 ranged from 0.56 to 0.57. Given these calculations, we sought to have at least 404 participants complete the trial. Based on our previous studies in Atlanta, we anticipated 20% loss to follow-up from the 504 enrolled participants. This sample size provides an estimated >99% power to detect a statistically significant contrast for aim 1 across a wide range of possible event-level pleasure scores.

#### Data Analysis

The planned primary analysis of aims is described in Table 1. Aim 1 involves the pair-wise comparison of pleasure scores between fitted and standard condoms. A linear mixed effects model with random effects for person and including arm, condom type, crossover period, and an arm*condom type interaction term will be conducted to account for repeated measures within participants (ie, the crossover design) and for repeated measures on coital acts within each of the 3 conditions. Model-based estimates and confidence intervals of the difference in pleasure score will be used to compare fitted and standard condoms. Additional control for participant-, partner-, and event-level correlates of pleasure in the above model will be considered in secondary analyses.

The primary analysis of aim 2 will be conducted at the participant-level using binary preference responses for comparison of fitted and standard condoms, collected at the final study visit (Table 1). For aim 2, we will assess whether a majority of participants preferred fitted over standard condoms using a logistic regression model with preference as the outcome and arm and crossover period as covariates. A confidence interval around the estimated probability of fitted condom preference will be computed.

A descriptive assessment for aim 3 will consist of calculating the per–anal sex act clinical failure proportion for the 3 condom conditions by dividing the number of total clinical failures by the total number of acts contributed for each condom type by participants in the MSM arm of the study. We will assess whether the proportion of failure for each condom type is below the threshold value that is to be determined by FDA. In order to adjust for study design, failure will also be assessed with a logistic mixed effects model with random effects for person with arm, condom type, crossover period, and an arm*condom type interaction term. For aim 4, we will use the logistic mixed model described in aim 3 to assess the odds of failure for fitted versus standard condoms within the MSM arm. Instances of anal sex among MSW will not be included in primary analyses because anal sex events occur frequently at the lifetime level for MSW but infrequently at monthly and even yearly levels [37]. This indicates lower levels of experience with this type of sex for many MSW, an issue that could introduce bias into study outcome assessment.

**Table 1:** Outcome measures used to assess each study aim.

Aim number and description		Outcome measure
1	To compare fitted condoms with standard condoms regarding levels of reported pleasure as determined by rating per condom use event	Pleasure-scale score (response item mean) for fitted condoms and standard condoms following each coital event
2	To compare fitted condoms with standard condoms regarding preference as determined by dichotomous preference among the 2 conditions at the study conclusion	Binary preference of fitted versus standard condoms at final study visit
3	To assess for fitted, thin, and standard condoms the total clinical failure rate of each type of condom for anal sex among MSM^a^ relative to a cut-point to be determined by the FDA^b^	Binary occurrence of clinical failure for each type of condom at each coital event
4	To compare fitted condoms with standard condoms regarding total clinical failure for anal sex	Binary occurrence of clinical failure for fitted and standard condoms at each coital event

^a^MSM: men who have sex with men.

^b^FDA: US Food and Drug Administration.

### Data Procedures

Study data collection was predominantly electronic and conducted through the study CDMS. The study used the Dacima Clinical Suite CDMS platform (Dacima Software Inc) compliant with all relevant FDA standards. For all office visits, eCRFs and the coital log were conducted on the CDMS. The study CDMS was a Web-based application, allowing participants to complete electronic coital logs at home with any device with an up-to-date Web browser. Information collected during recruitment and phone screenings was not collected using the CDMS but instead was collected through an electronic survey system, SurveyGizmo (covered by a Health Insurance Portability and Accountability Act business associate agreement) and transferred to a secure Emory database that allowed for potential participants to be contacted regarding the study. None of the data collected during recruitment and phone screenings was used as part of the study dataset. For coital log entries, participants used a secure link to access study forms that required a log-in with username and password protection. Details on methods for data quality assurance and laboratory procedures are in Multimedia Appendix 2.

## Results

The study assessed a total of 13,524 individuals for phase 1 eligibility through field-based screening. Of the 2819 initially eligible individuals, 1037 (36.8%) completed phase 2 eligibility assessment by phone; 681 were eligible and 542 attended a baseline visit. Of the 542 who attended the baseline visit, 504 were enrolled in the trial (93%). Baseline demographic and behavioral characteristics of study participants, by study arm and study condom type, are described in Table 2. MSM and MSW were similar across many traits such as race and ethnicity, marital status, portion circumcised, self-rated condom experience, erectile function, and condom failure in the last 6 months. Men in the MSM arm were older, with 47% (119/252) over the age of 30 years compared to 19% (48/252) of MSW being over 30 years. Fewer MSM were students than MSW (11% [28/252] and 53% [133/252], respectively), likely an artifact of recruitment. Nearly three-quarters of participants in both arms rated themselves as very experienced with using condoms (186/252 MSM and 187/252 MSW), the majority had used condoms in the past 30 days, and just over one-third in each arm reported condom failure in the last 6 months (81/252 MSM and 86/252 MSW).

**Table 2:** Baseline demographic and behavioral characteristics of study participants.

Characteristics				Participant strata				Condom type used					
				MSM^a^ (n=252), n (%)		MSW^b^ (n=252), n (%)		Blue (n=464), n (%)		Black (n=468), n (%)		Yellow (n=469), n (%)	
**Demographics**												
	**Age at baseline in years**											
		< 20		10 (4)		46 (18)		53 (11)		50 (11)		50 (11)
		20-24		54 (21)		104 (41)		146 (31)		149 (32)		151 (32)
		25-29		69 (27)		54 (21)		110 (24)		113 (24)		111 (24)
		30-39		71 (28)		37 (15)		98 (21)		100 (21)		100 (21)
		40-54		48 (19)		11 (4)		57 (12)		56 (12)		57 (12)
	**Race and ethnicity**											
		Hispanic		31 (12)		31 (12)		56 (12)		57 (12)		57 (12)
		White non-Hispanic		122 (48)		119 (47)		217 (47)		219 (47)		220 (47)
		African-American non-Hispanic		79 (31)		52 (21)		125 (27)		124 (27)		126 (27)
		Other non-Hispanic		20 (8)		49 (20)		65 (14)		67 (14)		65 (14)
		Prefer not to answer, non-Hispanic		0 (0)		1 (0)		1 (0)		1 (0)		1 (0)
	**Sexual identity**											
		Homosexual/gay		228 (90)		0 (0)		204 (44)		210 (45)		205 (44)
		Bisexual		21 (8)		5 (2)		24 (5)		25 (5)		24 (5)
		Heterosexual/straight		1 (0)		245 (97)		234 (50)		231 (49)		236 (50)
		Other		2 (1)		2 (1)		2 (0)		2 (0)		4 (1)
	**Education**											
		College, postgraduate, or professional school		160 (63)		116 (46)		253 (55)		254 (54)		256 (55)
		Some college, associate’s degree, or technical school		71 (28)		80 (32)		138 (30)		141 (30)		142 (30)
		High school, GED^c^, or less		21 (8)		56 (22)		73 (16)		73 (16)		71 (15)
	**Income**											
		<$20,000		62 (25)		85 (34)		136 (29)		136 (29)		138 (29)
		$20,000-$29,999		35 (14)		24 (10)		54 (12)		55 (12)		56 (12)
		$30,000-$39,999		27 (11)		17 (7)		42 (9)		43 (9)		40 (9)
		$40,000-$49,999		28 (11)		18 (7)		42 (9)		42 (9)		44 (9)
		≥50,000		90 (36)		85 (34)		159 (34)		160 (34)		160 (34)
		Don't know		10 (4)		23 (9)		31 (7)		32 (7)		31 (7)
	**Marital status, current**											
		Legally married/registered domestic partnership/civil union		19 (8)		26 (10)		41 (9)		42 (9)		43 (9)
		Divorced/separated		11 (4)		8 (3)		18 (4)		17 (4)		18 (4)
		Never married		222 (88)		218 (87)		405 (87)		409 (87)		408 (87)
	**Employment**											
		Employed		203 (81)		111 (44)		284 (61)		291 (62)		285 (61)
		Student		28 (11)		133 (53)		152 (33)		150 (32)		155 (33)
		Unemployed/retired/other		21 (8)		8 (3)		28 (6)		27 (6)		29 (6)
	**Homeless, last 6 months**											
		Yes		8 (3)		7 (3)		13 (3)		12 (3)		12 (3)
		No		244 (97)		245 (97)		451 (97)		456 (97)		457 (97)
**Sex history**												
	**Circumcised**											
		Circumcised (cut)		207 (82)		211 (84)		385 (83)		385 (82)		387 (83)
		Uncircumcised (uncut)		45 (18)		41 (16)		79 (17)		83 (18)		82 (17)
	**Number of insertive anal sex partners (MSM) or vaginal sex partners (MSW), past 30 days**												
		1		129 (51)		206 (82)		307 (66)		311 (66)		314 (67)
		2		56 (22)		35 (14)		85 (18)		87 (19)		86 (18)
		3		40 (16)		9 (4)		45 (10)		46 (10)		45 (10)
		≥4		27 (11)		2 (1)		27 (6)		24 (5)		24 (5)
	**Erectile function scale, with condom, past 6 months^d^**												
		No erectile dysfunction		157 (62)		186 (74)		323 (70)		317 (68)		323 (69)
		Mild erectile dysfunction		57 (23)		39 (15)		84 (18)		90 (19)		90 (19)
		Moderate to severe erectile dysfunction		9 (4)		1 (0)		8 (2)		10 (2)		8 (2)
		Missing		29 (12)		26 (10)		49 (11)		51 (11)		48 (10)
	**Used the following (choose all that apply)**												
		Pill such as Viagra, Cialis, or Levitra		28 (11)		2 (1)		29 (6)		28 (6)		29 (6)
		Testosterone		5 (2)		1 (0)		6 (1)		6 (1)		6 (1)
		Injection into your penis to get an erection		1 (0)		0 (0)		1 (0)		1 (0)		1 (0)
		Vacuum or penis pump to get an erection		3 (1)		0 (0)		3 (1)		2 (0)		3 (1)
		Penile implant		0 (0)		0 (0)		0 (0)		0 (0)		0 (0)
		None of the above		219 (87)		245 (99)		426 (93)		431 (93)		431 (93)
		Missing		1 (0)		4 (2)		4 (1)		4 (1)		4 (1)
**Condom use and failure, at baseline**													
	**Used a condom for insertive anal sex (MSM) or vaginal sex (MSW), past 30 days**												
		Yes		181 (72)		201 (80)		358 (77)		353 (75)		358 (76)
		No		56 (22)		40 (16)		84 (18)		92 (20)		90 (19)
		Missing		15 (6)		11 (4)		22 (5)		23 (5)		21 (4)
	**Self-rated condom experience**												
		Not very experienced		11 (4)		6 (2)		17 (4)		17 (4)		17 (4)
		Somewhat experienced		55 (22)		59 (23)		105 (23)		108 (23)		105 (22)
		Very experienced		186 (74)		187 (74)		342 (74)		343 (73)		347 (74)
	**Rating of length of last condom used, measured at baseline**												
		Very good		36 (14)		53 (21)		82 (18)		83 (18)		82 (17)
		Good		85 (34)		105 (42)		179 (39)		175 (37)		179 (38)
		Moderate		47 (19)		34 (13)		77 (17)		75 (16)		77 (16)
		Poor		12 (5)		9 (4)		19 (4)		19 (4)		19 (4)
		Very poor		1 (0)		0 (0)		1 (0)		1 (0)		1 (0)
		Missing		71 (28)		51 (20)		106 (23)		115 (25)		111 (24)
	**Rating of width of last condom used, measured at baseline**												
		Very good		28 (11)		44 (17)		66 (14)		67 (14)		67 (14)
		Good		73 (29)		99 (39)		166 (36)		165 (35)		166 (35)
		Moderate		49 (19)		44 (17)		86 (19)		81 (17)		85 (18)
		Poor		29 (12)		14 (6)		38 (8)		38 (8)		38 (8)
		Very poor		2 (1)		0 (0)		2 (0)		2 (0)		2 (0)
		Missing		71 (28)		51 (20)		106 (23)		115 (25)		111 (24)
	**Condom self-efficacy score^e^**											
		Scored below 16		116 (46)		130 (52)		226 (49)		229 (49)		227 (48)
		Scored 16		136 (54)		122 (48)		238 (51)		239 (51)		242 (52)
	**Started having sex without condom, then pulled out and put one on**												
		Yes		59 (23)		94 (37)		140 (30)		142 (30)		140 (30)
		No		164 (65)		132 (52)		275 (59)		275 (59)		281 (60)
		Missing		29 (12)		26 (10)		49 (11)		51 (11)		48 (10)
	**Started having sex with condom, then pulled out and took it off before sex was over**												
		Yes		77 (31)		72 (29)		138 (30)		141 (30)		138 (29)
		No		146 (58)		154 (61)		277 (60)		276 (59)		283 (60)
		Missing		29 (12)		26 (10)		49 (11)		51 (11)		48 (10)
	**Condom broke, slipped, or both during sex, past 6 months**												
		Yes		81 (32)		86 (34)		158 (34)		154 (33)		152 (32)
		No		142 (56)		140 (56)		257 (55)		263 (56)		269 (57)
		Missing		29 (12)		26 (10)		49 (11)		51 (11)		48 (10)

^a^MSM: men who have sex with men.

^b^MSW: men who have sex with women.

^c^GED: general education development.

^d^Erectile function scaled using the 5-item International Index of Erectile Function questionnaire [38].

^e^Condom self-efficacy scored using a 7-item scale adapted from previous work and with demonstrated evidence of internal reliability [39,40].

## Discussion

This protocol describes a blinded, crossover randomized clinical trial designed to compare the performance of standard, thin, and fitted condoms. To our knowledge, this is the first clinical trial of condoms to include preference or pleasure as primary outcomes. Pleasure is an inherently ephemeral experience, and yet is essential to the sexual experience. Qualitative literature is rife with critiques of how condoms are perceived to limit pleasure. For instance, condom use has been described as similar to “eating candy with the wrapper on” in settings as diverse as Brazil [41], Tanzania [7], and the Philippines [42]. There is growing consensus that issues regarding pleasure and condoms merit consideration, exemplified by a Bill and Melinda Gates Foundation grant call directly addressing this issue by instigating funding to develop new condom innovations [43]. The call noted the many health benefits of condoms and that “the primary drawback...is that condoms decrease pleasure as compared to no condom, creating a trade-off that many men find unacceptable.” Explicitly incorporating pleasure into the primary aims and hypotheses of future clinical trials could, in many instances, be accomplished without substantial additional effort.

A key feature of the study design is recruitment of equal numbers of MSM and MSW. Studies assessing condom performance among MSM are merited due to disparate impact of HIV, with over 2 out of every 3 new HIV diagnoses in the United States in 2015 occurring among MSM [44]. Equal enrollment of MSM and MSW will allow for assessment of whether study condoms of all types have sufficiently low failure rates as to merit an FDA label indication for anal sex.

This study incorporated a number of practices to minimize potential bias of primary outcomes. To minimize recall bias, participants were provided with the electronic coital log to complete following sex acts. Automated daily electronic reminders encouraged participants to complete a coital log entry for any sex acts not previously reported. Another advantage of electronic data collection is that it allowed for show/hide and piping features that turned what would have been a confusing paper report form into a short series of simple, answerable questions. Survey logic enabled by electronic data collection also allowed for us to incorporate a methodological innovation to decrease misreporting of condom failure; the survey system autogenerated a message that provided a summary of participant’s self-report data, with an option to confirm it or to correct it. Given that a small proportion of incorrectly reported data (eg, 3%) could substantially influence the likely rare study outcome of condom failure, we view this data validation step as holding substantial potential value.

To minimize response and recall bias, financial incentives were provided for regular participant use of the coital log; the same incentive amount was provided for coital log reports of no sex as for coital log reports of using study condoms. In contrast, some past studies required participants to use all of a set of study condoms prior to receiving incentives at their next study visit [45,46], or participants were given additional incentives for reporting on each additional condom use [45]. Such incentives could lead to participant overreporting use of study product to enhance their ability to receive further incentives.

This study is subject to a number of limitations. There is no gold standard laboratory measurement of clinical condom failure, condom preference, or pleasure. Instead, study outcomes rely on self-report, which multiple reviews have identified as problematic for reporting of outcomes relating to sex [47,48]. We sought to mitigate potential areas of bias along lines suggested by these reviews, such as shorter periods of recall and measurement specific to a sexual act and partner. For measurement bias regarding pleasure and preference, such as these constructs being potentially subjective and challenging to quantify, we expect bias would be random given that the study is blinded and thus would bias toward the null hypothesis.

In conclusion, this study protocol describes a clinical trial of condoms that incorporates novel outcomes of pleasure and preference into the primary aims and uses a number of methods to minimize potential sources of bias. The trial includes outcomes for both MSM and MSW, allowing for enhanced understanding of condom performance among a key population. Trial results may inform FDA label indication for anal sex and provide new information regarding the relative performance of different types of condoms.

## Appendix

Multimedia Appendix 1 World Health Organization registration and extended information.

Multimedia Appendix 2 Text appendices.

Multimedia Appendix 3 Follow-up visit structure.

Multimedia Appendix 4 Baseline visit questionnaire.

## Abbreviations

CDMS: clinical data management system
CFR: US Code of Federal Regulations
eCRF: electronic case report form
FDA: US Food and Drug Administration
IRB: institutional review board
ISO: International Organization for Standardization
MSM: men who have sex with men
MSW: men who have sex with women
STD: sexually transmitted disease

## References

[1] Centers for Disease Control and Prevention (2018). Press release: New CDC analysis shows steep and sustained increases in STDs in recent years.

[2] Centers for Disease Control and Prevention (2018). HIV Surveillance Supplemental Report 23(1): Estimated HIV incidence and prevalence in the United States, 2010-2015.

[3] Johnson WD, O'Leary A, Flores SA (2018). Per-partner condom effectiveness against HIV for men who have sex with men. AIDS.

[4] Steiner MJ, Cates W, Warner L (1999). The real problem with male condoms is nonuse. Sex Transm Dis.

[5] Charania MR, Crepaz N, Guenther-Gray C, Henny K, Liau A, Willis LA, Lyles CM (2011). Efficacy of structural-level condom distribution interventions: a meta-analysis of U.S. and international studies, 1998-2007. AIDS Behav.

[6] Baker H, Fried A, Cloete A, Sigel C, Miranda D, Guillen J, Rochat R, Siegler A (2018). Give what the people want: a situational analysis of condom distribution and a feasibility study of user-friendly condoms in Cape Town, South Africa. J Assoc Nurses AIDS Care.

[7] Siegler AJ, Mbwambo JK, McCarty FA, DiClemente RJ (2012). Condoms “contain worms and “cause HIV” in Tanzania: Negative Condom Beliefs Scale development and implications for HIV prevention. Soc Sci Med.

[8] Frye V, Koblin B, Chin J, Beard J, Blaney S, Halkitis P, Vlahov D, Galea S (2010). Neighborhood-level correlates of consistent condom use among men who have sex with men: a multi-level analysis. AIDS Behav.

[9] Reece M, Herbenick D, Schick V, Sanders SA, Dodge B, Fortenberry JD (2010). Condom use rates in a national probability sample of males and females ages 14 to 94 in the United States. J Sex Med.

[10] Siegler AJ, Voux AD, Phaswana-Mafuya N, Bekker L, Sullivan PS, Baral SD, Winskell K, Kose Z, Wirtz AL, Stephenson R (2014). Elements of condom-use decision making among South African men who have sex with men. J Int Assoc Provid AIDS Care.

[11] Wechsberg WM, Luseno WK, Kline TL, Browne FA, Zule WA (2010). Preliminary findings of an adapted evidence-based woman-focused HIV intervention on condom use and negotiation among at-risk women in Pretoria, South Africa. J Prev Interv Community.

[12] Hernández-Romieu AC, Siegler AJ, Sullivan PS, Crosby R, Rosenberg ES (2014). How often do condoms fail? A cross-sectional study exploring incomplete use of condoms, condom failures and other condom problems among black and white MSM in southern USA. Sex Transm Infect.

[13] DePadilla L, Windle M, Wingood G, Cooper H, DiClemente R (2011). Condom use among young women: modeling the theory of gender and power. Health Psychol.

[14] Crosby RA, Yarber WL, Graham CA, Sanders SA (2010). Does it fit okay? Problems with condom use as a function of self-reported poor fit. Sex Transm Infect.

[15] Calabrese SK, Reisen CA, Zea MC, Poppen PJ, Bianchi FT (2012). The pleasure principle: the effect of perceived pleasure loss associated with condoms on unprotected anal intercourse among immigrant Latino men who have sex with men. AIDS Patient Care STDS.

[16] Carballo-Diéguez A, Ventuneac A, Dowsett GW, Balan I, Bauermeister J, Remien RH, Dolezal C, Giguere R, Mabragaña M (2011). Sexual pleasure and intimacy among men who engage in. AIDS Behav.

[17] Scott-Sheldon LAJ, Marsh KL, Johnson BT, Glasford DE (2006). Condoms + pleasure = safer sex? A missing addend in the safer sex message. AIDS Care.

[18] Reece M, Briggs L, Dodge B, Herbenick D, Glover R (2010). Perceptions of condom fit and feel among men living with HIV. AIDS Patient Care STDS.

[19] Reece M, Herbenick D, Dodge B (2009). Penile dimensions and men's perceptions of condom fit and feel. Sex Transm Infect.

[20] Reece M, Dodge B, Herbenick D, Fisher C, Alexander A, Satinsky S (2007). Experiences of condom fit and feel among African-American men who have sex with men. Sex Transm Infect.

[21] Grov C, Wells BE, Parsons JT (2013). Self-reported penis size and experiences with condoms among gay and bisexual men. Arch Sex Behav.

[22] Centers for Disease Control and Prevention (2018). HIV Surveillance Report, 2016.

[23] Golombok S, Harding R, Sheldon J (2001). An evaluation of a thicker versus a standard condom with gay men. AIDS.

[24] Reece M, Herbenick D, Sanders SA, Monahan P, Temkit M, Yarber WL (2008). Breakage, slippage and acceptability outcomes of a condom fitted to penile dimensions. Sex Transm Infect.

[25] (2016). Condom performance in a longitudinal enhanced assessment of user experiences (C-PLEASURE) (NCT02753842).

[26] (1995). Testing guidance for male condoms made from new material (non-latex).

[27] Reece M, Herbenick D, Schick V, Sanders SA, Dodge B, Fortenberry JD (2010). Sexual behaviors, relationships, and perceived health among adult men in the United States: results from a national probability sample. J Sex Med.

[28] Wall KM, Stephenson R, Sullivan PS (2013). Frequency of sexual activity with most recent male partner among young, Internet-using men who have sex with men in the United States. J Homosex.

[29] Centers for Disease Control and Prevention (2002). Approval of a new rapid test for HIV antibody. MMWR Morb Mortal Wkly Rep.

[30] (2012). Use and procurement of additional lubricants for male and female condoms: WHO/UNFPA/FHI360: advisory note.

[31] Herbenick D, Schick V, Reece M, Sanders SA, Smith N, Dodge B, Fortenberry JD (2013). Characteristics of condom and lubricant use among a nationally representative probability sample of adults ages 18-59 in the United States. J Sex Med.

[32] Groves R, Fowler F, Couper M, Lepkowski J, Singer E, Tourangeau R (2004). Survey Methodology. 2nd Edition.

[33] Horvath KJ, Beadnell B, Bowen AM (2007). A daily web diary of the sexual experiences of men who have sex with men: comparisons with a retrospective recall survey. AIDS Behav.

[34] Siegler AJ, Boos E, Rosenberg ES, Cecil MP, Sullivan PS (2018). Validation of an Event-Level, Male Sexual Pleasure Scale (EMSEXpleasure) among condom-using men in the US. Arch Sex Behav.

[35] International Organization for Standardization (2012). Condoms—guidance on clinical studies—Part 1: male condoms, clinical function studies based on self-reports. ISO.

[36] Sullivan PS, Rosenberg ES, Sanchez TH, Kelley CF, Luisi N, Cooper HL, Diclemente RJ, Wingood GM, Frew PM, Salazar LF, Mulligan MJ, Peterson JL (2015). Explaining racial disparities in HIV incidence in black and white men who have sex with men in Atlanta, GA: a prospective observational cohort study. Ann Epidemiol.

[37] Herbenick D, Reece M, Schick V, Sanders SA, Dodge B, Fortenberry JD (2010). Sexual behavior in the United States: results from a national probability sample of men and women ages 14-94. J Sex Med.

[38] Rosen RC, Riley A, Wagner G, Osterloh IH, Kirkpatrick J, Mishra A (1997). The international index of erectile function (IIEF): a multidimensional scale for assessment of erectile dysfunction. Urology.

[39] Wingood GM, DiClemente RJ (1998). Partner influences and gender-related factors associated with noncondom use among young adult African American women. Am J Community Psychol.

[40] Crosby RA, DiClemente RJ, Salazar LF, Wingood GM, McDermott-Sales J, Young AM, Rose E (2013). Predictors of consistent condom use among young African American women. AIDS Behav.

[41] de Bessa GH (2006). Ethnophysiology and contraceptive use among low-income women in urban Brazil. Health Care Women Int.

[42] Lucea MB, Hindin MJ, Gultiano S, Kub J, Rose L (2013). The context of condom use among young adults in the Philippines: implications for HIV prevention. Health Care Women Int.

[43] Bill annd Melinda Gates Foundation (2013). Global Grand Challenges: Develop the next generation of condom (Round 11).

[44] Centers for Disease Control and Prevention HIV Surveillance Report, 2015.

[45] Macaluso M, Blackwell R, Jamieson DJ, Kulczycki A, Chen MP, Akers R, Kim D, Duerr A (2007). Efficacy of the male latex condom and of the female polyurethane condom as barriers to semen during intercourse: a randomized clinical trial. Am J Epidemiol.

[46] Beksinska M, Smit J, Mabude Z, Vijayakumar G, Joanis C (2006). Performance of the Reality polyurethane female condom and a synthetic latex prototype: a randomized crossover trial among South African women. Contraception.

[47] Taylor D (2009). Issues in the design, analysis and interpretation of condom functionality studies. Contraception.

[48] Noar SM, Cole C, Carlyle K (2006). Condom use measurement in 56 studies of sexual risk behavior: review and recommendations. Arch Sex Behav.

