# Supplementary material for: Double-Blind, Single-Center, Randomized Three-Way Crossover Trial of Fitted, Thin, and Standard Condoms for Vaginal and Anal Sex: C-PLEASURE Study Protocol and Baseline Data
Source: JMIR Res Protoc. 2019 Apr 23;8(4):e12205. doi: 10.2196/12205 (PMC6658242; doi:10.2196/12205)
Supplement: Multimedia Appendix 1 [file resprot_v8i4e12205_app1.docx]

**Contents**

1. WHO Trial Registration Data Set
2. Data Monitoring Committee
3. Access to Data
4. Study Aims and Other Areas of Exploratory Research and Interest
5. Recruitment Procedures
6. Study Product
7. Randomization and blinding procedures
8. Participant Compensation
9. Training
10. Safety monitoring and adverse event reporting

**I. WHO Trial Registration Data Set**

| 1. Primary registry and trial identifying number | ClinicalTrials.gov NCT02753842 |
| --- | --- |
| 2. Date of registration in primary registry | 28 April 2016 |
| 3. Secondary identifying numbers | NIH SBIR Grant: R44 HD078154  Emory IRB: IRB00083754 |
| 4. Source(s) of monetary or material support | Small Business Innovation Research grant from the National Institutes of Health (NIH) |
| 5. Primary sponsor | National Institute of Child Health and Human Development, National Institutes of Health (NIH) |
| 6. Secondary sponsor(s) | none |
| 7. Contact for public queries | Aaron Siegler, asiegle@emory.edu |
| 6. Contact for scientific queries | Aaron Siegler, asiegle@emory.edu |
| 7. Public title | A Double-Blind, Single Center, Randomized 3-way Crossover Trial to Determine Pleasure for Fitted, Thin and Standard Condoms, and to Assess Clinical Failure for Vaginal and Anal Sex |
| 8. Scientific title | A Double-Blind, Single Center, Randomized 3-way Crossover Trial to Determine Pleasure for Fitted, Thin and Standard Condoms, and to Assess Clinical Failure for Vaginal and Anal Sex  Short Title: Condom Performance in a Longitudinal Enhanced ASsessment of UseR Experiences (C-PLEASURE) |
| 9. Countries of recruitment | United States |
| 10. Health condition(s) or problem(s) studied | 1. Event-level sexual pleasure scale score [ Time Frame: Up to 12 weeks ] 2. Condom preference [ Time Frame: Up to 12 weeks ] 3. Clinical condom failure for anal sex, all condoms below FDA cut-point [ Time Frame: Up to 12 weeks ] 4. Clinical condom failure for anal sex, fitted compared to standard condoms [ Time Frame: Up to 12 weeks ] |
| 11. Intervention(s) | 1. Fitted condoms: Fitted condoms are available in 56 sizes based on combinations of length (approximately 10mm increments) and circumference (approximately 2 mm increments). Fitted condom thickness is 70 microns ± 10 microns. Appropriate condom size is user-determined with a paper template fitting tool graduated with non-sequential numbering and lettering for maximum privacy.  Participants will receive 5 fitted condoms in plain aluminum foils, identifiable only by a two-digit number printed on the foil. Participants will also receive ten plain foil packets of 10ml commercially available condom-compatible water-based lubricant.  2.Thin Condoms: Thin condoms have dimensions of 185mm ± 10mm length, 53mm ± 2mm width, and 50 microns ± 5 microns thick.  Participants will receive 5 thin condoms in plain aluminum foils, identifiable only by a two-digit number printed on the foil. Participants will also receive ten plain foil packets of 10ml commercially available condom-compatible water-based lubricant.  3. Standard Condoms: Standard condoms have dimensions of 185mm ± 10mm length, 53mm ± 2mm width, and 70 microns ± 10 microns thick.  Participants will receive 5 thin condoms in plain aluminum foils, identifiable only by a two-digit number printed on the foil. Participants will also receive ten plain foil packets of 10ml commercially available condom-compatible water-based lubricant. |
| 12. Key inclusion and exclusion criteria | Inclusion criteria:  1. Age 18-54  2. Lives in or near Atlanta metropolitan statistical area  3. Plans to be in Atlanta for the majority of the 12 weeks of enrollment  4. Able to independently complete survey instruments in English  5. Male sex at birth  6. Currently identifies as male  7a. For MSM, self-report to have only had sex with men in the past four weeks  7b. For MSW, self-report to have only had sex with women in the past four weeks,  8. Self-report no transgender sex partners in the past four weeks,  9a. For MSM, self-report at least one anal sex act in the past four weeks,  9b. For MSW, self-report at least one vaginal sex act in the past four weeks,  10a. For MSM, self-report intends to have sex only with men in the next 12 weeks,  10b. For MSW, self-report intends to have sex only with women in the next 12 weeks,  11. For MSM, self-reports an insertive role in the past four weeks or willing to be the insertive partner when using study condoms for anal sex,  12. Willing and able to have sex using a latex condom provided by study,  13. For MSW, report that current partner is not currently pregnant,  14. For MSW, report that current partner does not desire to become pregnant currently or in the next 12 weeks,  15. Consistently able to maintain an erection while using condoms,  16. Not allergic to latex,  17. Current partner(s) not allergic to latex  18. No genital piercings  19a. For MSW, female current partner(s) does (do) not have vaginal piercings  19b. For MSM, male current partner(s) does (do) not have anal piercings  20. Current partner(s) not known to be HIV-positive,  21. Self-report absence of sexually transmitted infections, including HIV  22. Willing to provide at least two means of contact  23. Willing to only use lubricant provided by study  24. Not allergic to water-based lubricant,  25. Current partner(s) not allergic to water-based lubricant,  26. Negative HIV rapid test result or confirmed negative HIV test at baseline  27. Willing to use a fitting tool to determine penile dimensions and report results to study staff. |
|  | Exclusion criteria:  1. <18 years of age or >54 years of age  2. Does not live in or near Atlanta MSA  3. Does not plan to be in Atlanta for the majority of the 12 weeks of enrollment  4. Unable to independently complete survey instruments in English  5. Not male sex at birth  6. Does not currently identify as male  7. Self-report to have had sex with both men and women in the past four weeks  8. Self-report transgender sex partners in the past four weeks  9. Self-report no anal (MSM) or vaginal (MSW) sex act in the past four weeks  10. Self-report intends to have sex with both men and women in the next 12 weeks  11. Never the insertive partner for anal sex or not willing to be the insertive partner when using study condoms for anal sex  12. Not willing or unable to have sex using a latex condom provided by study  13. Plans to not have sex in the next four weeks  14. For MSW, report that current partner is currently pregnant  15. For MSW, report that current partner desires to become pregnant currently or in the next 12 weeks  16. Unable to consistently maintain an erection while using condoms  17. Allergic to latex  18. Current partner(s) allergic to latex  19. Genital piercings  20. For MSW, female current partner(s) has (have) vaginal piercings  21. For MSM, male current partner(s) has (have) anal piercings  22. Current partner(s) known to be HIV-positive  23. Self-report presence of sexually transmitted infections, including HIV  24. Confirmed HIV positive at baseline  25. Not willing to provide at least two means of contact  26. Not willing to only use lubricant provided by study  27. Allergic to water-based lubricant  28. Current partner(s) allergic to water-based lubricant  29. Not willing to use a fitting tool to determine penile dimensions |
| 13. Study type | Type: Interventional  Allocation: Randomized  Masking: Triple (Participant, Investigator, Outcomes Assessor)  Assignment: triple crossover, double arm  Purpose: Prevention |
| 14. Date of first enrollment | May 2016 |
| 15. Target sample size | 504 |
| 16. Recruitment status | May 2017 |
| 17. Primary outcome(s) | Clinical event-level condom failure, participant-rated pleasure (based on an event-level pleasure scale), participant condom preference (measured at final study visit) |
| 18. Key secondary outcomes | Pairwise comparisons of event-level pleasure scale by condom type, Pairwise comparisons of overall condom preference at study conclusion |

**II. Data Monitoring Committee**

Given the minimal-risk context of this study, there was no data monitoring committee.

**III. Access to Data**

PRISM Research at Emory University in Atlanta, Georgia will retain full access to the trial dataset.

**IV. Study Aims and Other Areas of Exploratory Research and Interest**

*Primary Study Aims*

1. To compare fitted condoms with standard condoms regarding levels of reported pleasure as determined by rating per condom use event.

- Hypothesis 1**.** Fitted condoms will have higher pleasure ratings than standard condoms.
- Hypothesis rationale 1. There is biological plausibility and published data indicating that condoms fitted to penile dimensions could improve perceptions of pleasure.

1. To compare fitted condoms with standard condoms regarding preference as determined by ranking of the two conditions at the study conclusion.

- Hypothesis 2. More participants will prefer fitted condoms than standard condoms.
- Hypothesis rationale 2. There is biological plausibility and published data indicating that condoms fitted to penile dimensions could improve perceptions of pleasure.

1. To assess for condoms (fitted, thin, standard) the total clinical failure rate of each type of condom for anal sex among MSM relative to a cut-point to be determined by the United States Food and Drug Administration (FDA).

- Hypothesis 3. All condom conditions will have clinical failure point estimates less than a cut-point level of acceptable clinical failure, with the cut-point to be determined by the FDA.
- Hypothesis rationale 3. Condoms are currently recommended for anal sex use for the prevention of STI and HIV by the U.S. Centers for Disease Control and Prevention (CDC), the United Nations, and the World Health Organization.

1. To compare fitted condoms with standard condoms regarding total clinical failure for anal sex among MSM.

- Hypothesis 4. Fitted condoms will have a lower total clinical failure rate than standard condoms for anal sex among MSM.
- Hypothesis rationale 4. Biological plausibility and published data from a prospective, event-level assessment of clinical failure indicate that fitted condoms may have superior clinical performance for anal sex [1].

## *Secondary Study Aims*

1. To compare thin condoms with standard condoms regarding levels of reported pleasure as determined by rating per condom use event.
2. To compare fitted condoms with thin condoms regarding levels of reported pleasure as determined by rating per condom use event.
3. To compare thin condoms with standard condoms regarding preference as determined by ranking of the two conditions at the study conclusion.
4. To compare fitted condoms with thin condoms regarding preference as determined by ranking of the two conditions at the study conclusion.

## *Other Areas of Exploratory Research Interest*

1. To assess differences in total clinical failure between fitted, thin, and standard condoms.
2. To analyze aims regarding pleasure/preference and fitted condoms (Aims 1 and 2) restricted to participants who received fitted study condoms outside standard sizes of width, length, and both width and length.
3. To analyze aims regarding pleasure/preference (Aims 1 and 2) restricted to participants in the top and bottom deciles of self-reported penis size.
4. To explore correlates of condom failure (type of condom, SES, level of condom experience).
5. To compare history of experiencing condom slippage and breakage with slippage and breakage experienced in the study period.
6. To perform visual analysis of condom breaks, conducted according to ISO Guidance Document, ISO TC 157 N 770,[^17^](#_ENREF_17) and correlates of breakage types.
7. To compare a baseline measure of perceptions of pleasure during condom use to event-level pleasure reported in the coital logs.
8. To compare previous measures of pleasure (history of pleasure using condoms) to pleasure prospectively reported during the study.
9. To assess whether there were differential changes in pleasure for certain individuals: those with history of erectile dysfunction or sexual performance issues; participants with certain penile dimensions; MSM vs. MSW.
10. To understand the clinical failure rate when a single condom is used for multiple types of sex (oral, anal, and/or vaginal), and correlates of this type of condom usage.
11. To compare history of erectile dysfunction and sexual performance issues to consistency of condom use.
12. To assess effect modification of study arm (i.e., MSM vs. MSW) on the relationship between study condition and outcome measure (i.e., pleasure or preference) for Aims 1 and 2.
13. To understand willingness and acceptability to use condoms, and reasons why participants do not use condoms through a comparison willingness at baseline to willingness at the end of the study period.
14. To understand the stability of condom preferences over time (prior to beginning the third crossover condition and at the end of the study).

**V. Recruitment Procedures**

Primary recruitment venues included bars, dance clubs, retail stores, street corners, restaurants, churches, college campuses and other public places. At the recruitment event, trained recruiters approached potential participants, obtained permission to screen, and administered a brief recruitment script and set of screening questions using an electronic handheld device. Recruiters handed out study contact cards, commercially-available condoms (not fitted), and lubricant. Eligible potential participants that completed the Stage 1 screening questions were sent an electronic consent form that indicated willingness to receive a full eligibility screening.

Flyers used in secondary recruitment efforts advertised the study in venues that men frequent, such as community and clinical spaces, colleges and universities (e.g., cafes, clubs, bars/restaurants, gyms, HIV/STI testing facilities, doctors’ offices, and sex shops). We also created paid, online advertisements in popular social networking sites including facebook.com, twitter.com, geosocial networking sites, and local news and culture sites. Flyers and online advertisements included a link to an online version of the Stage 1 screening questions and an electronic consent to receive a full eligibility screening. We also recruited participants who had consented to be contacted from previous research projects conducted by the research team in Atlanta.

**VI. Study Product**

Standard condoms were made with dimensions of 185mm ± 10mm length, 53mm ± 2mm width, and 70 microns ± 10 microns thick. Standard condoms were selected as the control because they are the most widely used condoms internationally due to their use by programs distributing condoms, such as USAID (Steven Hamel, BS, email communication, June 23, 2015).

Thin condoms were made with dimensions of 185mm ± 10mm length, 53mm ± 2mm width, and 50 microns ± 5 microns thick. Thin condoms were selected as an additional study condom because they are widely sold condoms in the United States and because of the general perception that they are more pleasurable to use than standard condoms.

Fitted condoms were made in 56 condom sizes based on combinations of length (approximately 10 mm increments) and circumference (approximately 2 mm increments), in dimensions cleared for use by FDA [2]. Thickness of fitted condoms was 70 microns ± 10 microns. To determine appropriate condom size, participants used a paper template graduated with non-sequential numbering and lettering.

Condoms were manufactured using identical latex formulation and silicone lubricants (approximately 400mg per condom), and with similar parallel wall designs. All condoms were manufactured by Karex Berhad according to specifications provided by the study investigators and provided in plain foil wrapping. Additionally, all references to branding were removed from the fitting tool and from any other study materials.

**VII. Randomization and blinding procedures**

The randomization process was conducted as follows: Within each study arm (MSM or MSW), an unblinded data coordinator developed permuted block randomization sequences in an electronic clinical data management system (CDMS). The block sizes were randomly chosen (e.g., 6, 12). These randomization sequences were stored in the CDMS. Following successful completion of baseline study visit procedures and self-measurement for fitted condoms, the participant was then automatically randomized by receiving the next available crossover condition assignment in the sequence within that participant’s arm. The CDMS then indicated to study staff which crossover condition to distribute at the baseline visit (or to mail if sizing occurred at home after the baseline visit) and at appropriate subsequent study visits.

As part of the blinding scheme, the different condom types were matched to three different colors. These colors were used to guide the process of condom distribution in the study. The colors, rather than the fitted, thin, and standard condom nomenclature, were the data entered into the clinical trial system. Study staff who were not involved in the assessment of outcomes or analyses were unblinded as needed to dispense the appropriate condoms to participants, but were trained in how to interact with blinded participants without disclosing allocations or otherwise influencing participants.

The study team recognizes an inherent limitation to the blinding scheme, in that some participants may have been able to detect their assigned condition. We sought to mitigate this potential bias by recording the perceived study condom assigned, and will seek to assess in secondary analyses whether participants who were able to detect their assigned study condition reported differently on key outcomes than participants who were unaware of their study condition.

**VIII. Participant compensation**

Participants were reimbursed for their time and effort in this study. Participants who attended a baseline enrollment visit were compensated $50 for the visit regardless of their eligibility or future study participation. Participants were reimbursed $35-$50 for each follow-up visit attended. Reimbursement amount depended on completion of coital logs. Participants who completed at least 10 coital logs (which could be marked as either affirmative or negative for using a study condom in the time since last report) in the two-week period were compensated $50 at their follow-up visit. Participants who did not complete 10 coital log entries were compensated $35 at their follow-up visit.

**IX. Staff and Participant Training**

Study recruiters completed a two-hour training on recruitment procedures and use of study screening instruments. Study staff interacting with participants, including recruiters, counselors, phlebotomists, and others involved with research events completed CITI training regarding biomedical and social/behavioral ethics in study operations, data management, and good clinical practice. All study staff involved in the handling of biological specimens were trained in Biohazard Safety. Study staff who had contact with blinded participants were trained in how to interact with blinded participants. Study staff who conducted HIV counseling and testing completed a three-day training in HIV prevention counseling and rapid testing, according to the CDC training curriculum and completed at least 40 hours of supervised HIV counseling and testing experience prior to independent counseling. Participants were retrained in correct condom use if they had one of the following issues in the previous study period: using condoms for more than one sex act, MSM not using lubricant during anal sex, using oil-based lubricant, using non-study lubricant, using lubricant on the inside of condom, or having multiple failures within the crossover period.

**X.** **Safety monitoring and adverse event reporting**

A safety and monitoring plan was in place which detailed instructions for data monitoring by the principal investigator, review of adverse event reports by the independent study clinician, and reporting of any adverse events to the Emory University IRB. All serious adverse events were reported to the Emory University IRB.

**References**

1. Reece M, Herbenick D, Sanders SA, Monahan P, Temkit M, Yarber WL. Breakage, slippage and acceptability outcomes of a condom fitted to penile dimensions. Sexually transmitted infections. 2008;84(2):143-149.
2. U.S. Food and Drug Administration Center for Devices and Radiological Health. TheyFit Male Condom 510(k) Approval No. K150072. <https://www.accessdata.fda.gov/cdrh_docs/pdf15/K150072.pdf2015>.
