# Supplementary material for: Double-Blind, Single-Center, Randomized Three-Way Crossover Trial of Fitted, Thin, and Standard Condoms for Vaginal and Anal Sex: C-PLEASURE Study Protocol and Baseline Data
Source: JMIR Res Protoc. 2019 Apr 23;8(4):e12205. doi: 10.2196/12205 (PMC6658242; doi:10.2196/12205)
Supplement: Multimedia Appendix 2 [file resprot_v8i4e12205_app2.docx]

**Appendix Section 1. Participant retention, withdrawal, and temporary holds**

A number of procedures implemented by study staff sought to minimize loss-to-follow-up. For instance, staff explained the study and procedural requirements during the informed consent process and reemphasized them at each study visit. Staff used SMS, telephone, and email reminder mechanisms prior to scheduled visits, with modality based on recorded participant preference. For participants who missed visits, initial contact attempts were made for immediate follow-up and rescheduling, as well as monthly attempts to reengage participants who had been unresponsive to previous scheduling attempts. To reduce the burden of study visit attendance on participants who did not own a vehicle, we made prepaid fare for a car service available for transit to study visits.

Participants were able to voluntarily withdraw from the study for any reason at any time. The study investigators could also withdraw participants from the study in order to protect participant or staff safety.

**Appendix Section 2.** **Crossover condition combinations for each study arm**

In this closed label, blinded trial, study condoms were manufactured in plain foil packaging, with identifying two-digit random codes printed on each foil. Blinding of study staff was role-based. The study statistician and the principal investigator, who are responsible for analyses and reporting results to FDA, will be blinded until after the initial analysis of study results has been conducted. Participants were also blinded in order to minimize bias. Further information around the randomization and blinding procedures is available in Additional file 1.

**Appendix Section 3. HIV Testing**

Participants received HIV counseling, rapid testing, and test results at their baseline visit per CDC guidelines [1]. Participants with preliminary positive results were scheduled to receive confirmatory HIV testing. All preliminary positive results were confirmed with a 4th generation immunoassay with reflex by a study laboratory per CDC guidelines [2]. Participants with preliminary positive rapid HIV tests received their incentive and their study visit ended at that time. The results of the confirmatory testing were returned by study staff experienced in HIV care linkage. Participants with confirmed HIV infection were ineligible to continue in the prospective study. The Georgia State Health Department was notified of any confirmed HIV-positive results in accordance with the law, a procedure that was explained to participants at consent. Participants with two invalid INSTI tests or who were unable to provide enough blood for the INSTI test were scheduled to receive confirmatory testing and paused from study participation until results were delivered. Participants who were confirmed to be negative for HIV infection were contacted to return to complete baseline visit procedures.

**Appendix Section 4. Participant Training**

Study staff trained all eligible participants regarding correct condom use based on a training module from a CDC-determined Effective Behavioral Intervention [3]. This training included condom application and removal, appropriate use (e.g. one condom per sex act), only using study-provided lubricant, and only applying lubricant to the exterior of the condom. MSM were instructed to use lubricant with all anal sex acts, MSW were instructed to use lubricant as needed or desired.

Participants also received training on accessing and completing the home electronic coital log. Study staff told participants to complete the log as soon as possible following intercourse, as well as in response to daily reminders and demonstrated how to access the log on their phones. Participants provided a preferred time for the daily coital log reminder, and this information was entered into the scheduling system to be used as a personalized reminder time.

Participants who did not determine their fitted condom size at home received a reminder to provide the study with their fitted condom size. Participants were instructed to return unused study condoms. Participants were also instructed to return any condoms that broke at their next follow-up visit in study-provided biohazard bags, as specified in ISO Guidance Document, ISO TC 157 N 770 [4].

**Appendix Section 5. Visit adverse event screening**

Study staff screened participants for symptoms of a STI or acute HIV infection at each follow-up visit. If the participant reported symptoms of any STI, study staff referred the participant for testing and treatment. In cases where the participant or study staff were unsure about the presence of STI symptoms, the participant was assessed by the study clinician and referred for testing and treatment as needed. Participants symptomatic for STIs received their incentive for the visit and were study stopped. This was recorded as an adverse event. If participants self-reported symptoms of acute HIV infection and either (a) had unprotected anal sex with a man or (b) had injected a substance not prescribed to the individual in the past 30 days, study staff were instructed to arrange for a blood draw for an HIV viral load test to assess for potential acute infection, provide participants their incentive and end their study visit. Upon receipt of HIV laboratory results, staff were to allow the participant to return to complete their study visit if there was no detectable viral load. If a participant had a detectable viral load, indicating acute HIV infection, study staff were instructed to link the participant to HIV care, study stop the participant, and record this as an adverse event.

Study staff determined whether participants or their partners had experienced any discomfort or issues related to the study condoms based on a series of standard questions. If any issues were communicated, study staff were to ask participants to have a picture taken at the site of the reaction to allow for the study clinician to provide a remote assessment. Depending on the severity of issues, as determined by the clinical judgment of the study physician, the participant would be study stopped. MSW participants were asked whether their partner had become pregnant. If that participant indicated that their partner was pregnant, and the expected date of conception was during the study or unknown, their partner was referred to the Grady Family Planning Wellness Clinic and study staff recorded this as an adverse event.

**Appendix Section 6. Ethics, consent and permissions**

Prior to study initiation the study sought and attained approval of the protocol, study documents, and informed consent process by the Emory University IRB. The principles of Informed Consent, according to FDA Regulations and International Conference on Harmonization (ICH) guidelines on Good Clinical Practice (GCP), were followed. Participant privacy and confidentiality was and will continue to be carefully protected through both staff training, study procedures, and data management practices. Study staff complied with all applicable local requirements to report communicable diseases identified among study participants to local health authorities. Participants were made aware of all reporting requirements during the study informed consent process.

**Appendix Section 7. Quality Assurance**

Monitoring visits were performed periodically by the principal investigator during the study to ensure that all aspects of the current, approved protocol/amendment(s) were followed. All eCRFs and electronic surveys were programmed into the CDMS, and included skip patterns to ensure that participant and staff responses to each question were logical based on responses to previous questions. Data entry fields included data validation and range limits, when appropriate, to ensure responses were logical. Study staff entering data could create and save queries in the CDMS on data forms to double check responses and source documents. Coital logs were reviewed by study staff with the participant at each follow-up visit to ensure accuracy and completion.

**Appendix Section 8. Laboratory testing and procedures**

At enrollment events, an FDA-approved HIV rapid test, the INSTI HIV-1 antibody test, (PMA number: BP090032/0) was used for HIV screening. Preliminary positive results were to be confirmed with a 4th generation immunoassay with reflex by a study laboratory per CDC recommendations (22). At both enrollment and follow-up visits, we assessed plasma viral load for participants who presented with symptoms that were indicative of acute HIV infection. We used the Abbott RealTime HIV-1 Assay, an in vitro reverse transcription-polymerase chain reaction (RT-PCR) assay, for viral load measurements on the automated m2000 System from plasma (range of detection: 40 to 10,000,000 copies/mL). Participants with preliminary positive HIV rapid test results had blood drawn and sent to a laboratory for confirmation testing. Participants who were symptomatic for acute HIV infection had blood drawn and sent to the study laboratory for viral load testing. The study staff adhered to standards of good clinical laboratory practice, and local standard operating procedures for specimen management including proper collection, processing, labeling, transport, and storage of specimens to the lab.

References:

1. Centers for Disease Control and Prevention. Approval of a new rapid test for HIV antibody. MMWR Morb Mortal Wkly Rep. 2002; doi:51(46):1051-1052.

2. Centers for Disease Control and Prevention, Association of Public Health Laboratories. Laboratory Testing for the Diagnosis of HIV Infection: Updated Recommendations. 2014. <http://stacks.cdc.gov/view/cdc/23447>. Accessed 22 Jun 2015.

3. Cicatelli Associates Incorporated. Implementation Manual, Focus on the Future: A brief, single-session intervention with young African American males who have sex with women, who report symptoms of an STD and/or have been diagnosed with an STD. 2012. <https://effectiveinterventions.cdc.gov/docs/default-source/fof-implementation-materials/FOF_Complete_IM_-_May_2012.pdf?sfvrsn=0>. Accessed 22 June 2015.

4. Condoms — Guidance on clinical studies — Part 1: Male condoms, clinical function studies based on self-reports. 2012. ISO TC 157 N 770.
