# Supplementary figures and images for: Double-Blind, Single-Center, Randomized Three-Way Crossover Trial of Fitted, Thin, and Standard Condoms for Vaginal and Anal Sex: C-PLEASURE Study Protocol and Baseline Data
Source: JMIR Res Protoc. 2019 Apr 23;8(4):e12205. doi: 10.2196/12205 (PMC6658242; doi:10.2196/12205)

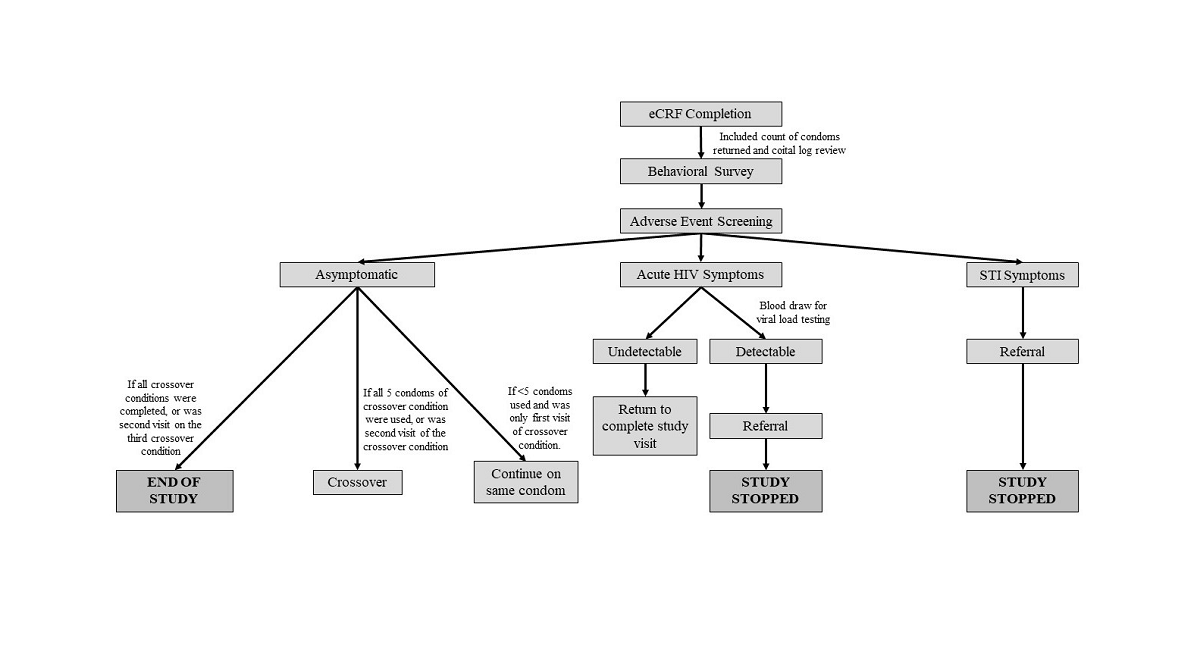

Supplement: Multimedia Appendix 3 [file resprot_v8i4e12205_app3.png]
