# Supplementary material for: Double-Blind, Single-Center, Randomized Three-Way Crossover Trial of Fitted, Thin, and Standard Condoms for Vaginal and Anal Sex: C-PLEASURE Study Protocol and Baseline Data
Source: JMIR Res Protoc. 2019 Apr 23;8(4):e12205. doi: 10.2196/12205 (PMC6658242; doi:10.2196/12205)
Supplement: Multimedia Appendix 4 [file resprot_v8i4e12205_app4.docx]

Baseline Survey

1/27/2016

[I. Demographics 2](#_Toc428351696)

[II. Sexual history 4](#_Toc428351697)

[III. Sexual dysfunction 6](#_Toc428351698)

[IV. Condom slippage and breakage 7](#_Toc428351699)

[V. Lubricant use 9](#_Toc428351700)

[VI. Therapeutic methods 10](#_Toc428351701)

[VII. Current condoms used 11](#_Toc428351702)

[VIII. Condom fit and feel 11](#_Toc428351703)

[IX. Self-efficacy around condom use 12](#_Toc428351704)

[X. Willingness to use condoms 12](#_Toc428351705)

[XI. HIV and STI history 12](#_Toc428351706)

[XII. Partner numbers and names in last 30 days 14](#_Toc428351707)

[XIII. Partner-specific questions 14](#_Toc428351708)

[XIV. Physical Sexual Pleasure 17](#_Toc428351709)

# Demographics

1. What is the highest level in school that you completed? [Source: NHBS Round 4; Element]
   1. College, post graduate, or professional school
   2. Some college, Associate’s degree and/or Technical School
   3. High school or GED
   4. Did not finish high school
   5. Don’t know
2. What best describes your employment status? Are you: [Source: Element, Adapted from NHBS Round 4 (DM6)]
   1. Employed full-time
   2. Employed part-time
   3. A full-time student
   4. A part-time student
   5. On active duty in US Armed Forces, Reserves, or National Guard
   6. Unable to work for health reasons
   7. Retired
   8. Unemployed
   9. Other
   10. Don’t know
   11. Refuse to Answer
3. What was your household income last year from all sources before taxes? (monthly/yearly) [Source: NHBS; Element]
   1. 0 to $417 (monthly) / 0 to $4,999 (yearly)
   2. $418 to $833 (monthly) / $5,000 to $9,999 (yearly)
   3. $834 to $1250 (monthly) / $10,000 to $14,999 (yearly)
   4. $1251 to $1667 (monthly) / $15,000 to $19,999 (yearly)
   5. $1668 to $2500 (monthly) / $20,000 to $29,999 (yearly)
   6. $2501 to $3333 (monthly) / $30,000 to $39,999 (yearly)
   7. $3334 to $4167 (monthly) / $40,000 to $49,999 (yearly)
   8. $4168 to $6250 (monthly) / $50,000 to $74,999 (yearly)
   9. $6251 or more (monthly) / $75,000 or more (yearly)
   10. Don’t know
4. Including yourself, how many people depended on this income? (must be at least 1) [Source: NHBS; Involvement, Element] (text numeric)
5. Do you rent, own, or stay at the place you sleep? [Source: Element]
   1. Rent
   2. Own
   3. Stay for nightly or monthly rate
   4. Stay for free
   5. Other arrangement
   6. Don’t know
6. In the past 6 months, have you been homeless at any time? *(By homeless, we mean you were living on the street, in a shelter, a Single Room Occupancy hotel (SRO), or living in a car?)* [Source: Involvement, Element]
   1. Yes
   2. No
7. Are you currently homeless? [Source: Involvement, Element] [show if previous question = “Yes”]
   1. Yes
   2. No
8. Do you currently have health insurance? *(This includes Medicare or Medicaid.)* [Source: NHBS, MMP; Element]
   1. Yes
   2. No
   3. Don’t know
9. Did you have health insurance at any time in the past 6 months? [Source: NHBS, MMP; Element] [show if current health ins = “No”]
   1. Yes
   2. No
10. The Affordable Care Act (‘Obamacare’ or ACA) gives a way for many Americans to buy private health insurance. This is done through Healthcare.gov, or the Health Insurance Marketplace.

Have you heard of this way to buy health insurance? [Source: Element] [show if current health ins = “No”]

- 1. Yes
  2. No

1. Thinking about why you have not gotten health insurance this way, do any of these reasons apply to you? *Check all that apply.* [Source: Element] [show if current health ins = “No” and heard of ACA = “Yes”]
   1. Multi-check
   2. Too busy to sign up
   3. Cost of the medical plans is too high
   4. Not sure how to sign up
   5. Do not qualify for these plans
   6. Might qualify for other programs, such as Medicaid or Medicare
   7. Not interested in having health insurance
   8. Other, please specify: ___________________
2. What kind of health insurance or coverage do you currently have? [Source: Involvement, Element] [show if Q16 = “Yes”]
   1. Private health insurance or HMO
   2. Medicaid
   3. Medicare
   4. TRICARE (CHAMPUS)
   5. Veterans Administration Coverage (VA)
   6. Other, please specify: ___________________
3. Do you get health insurance through your employer, a partner’s employer, or other family member’s employer? [Source Modified MMP 2015-2017; Element] [show if Q12 “Private health insurance or HMO”]
   1. Yes
   2. No
   3. Don’t know
4. Did you purchase this private health care insurance through Healthcare.gov, or the Health Insurance Marketplace? [Source Modified MMP 2015-2017; Element] [show if Q13 = “No” or “Don’t know”]
   1. Yes
   2. No
   3. Don’t know
5. Do you think of yourself as: [Source: Involvement, Element]
   1. Heterosexual or straight
   2. Homosexual or gay
   3. Bisexual
   4. Other, please specify: ___________________
6. Which of the following best describes your current marital status? [Source: Element]
   1. Legally married
   2. Registered domestic partnership or civil union
   3. Widowed
   4. Divorced
   5. Separated
   6. Never married
7. Is/was this partner: [Source: Element] [show if marital status ≠ “Never married”]
   1. Male
   2. Female
   3. ~~Transgender: male to female~~
   4. ~~Transgender: female to male~~

# Sexual history

Now we are going to ask you some personal questions about your sexual history.

1. Is your penis circumcised (cut) or uncircumcised (uncut)? [Source: Involvement, images from Plank et al., 2010, *AIDS Behav*]


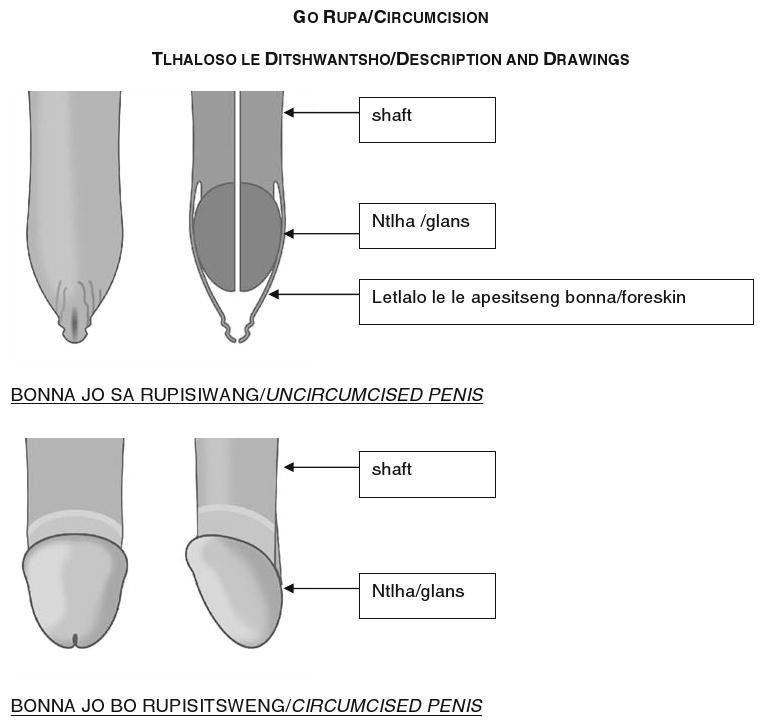


Circumcised (cut) penis

Uncircumcised (uncut) penis


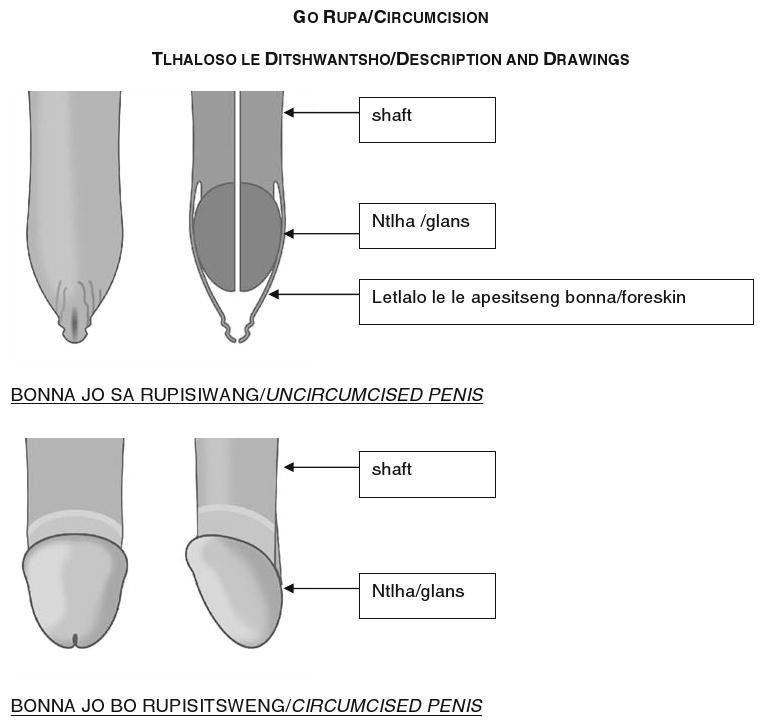


- 1. Yes
  2. No
  3. Don’t know

1. Which of the following have you ever done? Mark all that apply.
2. Had receptive anal sex with a man
3. Had insertive anal sex with a man
4. Had anal sex with a woman
5. Had vaginal sex with a woman
6. (If ever anal) How old were you the first time you had anal sex? (Drop down with ages)
7. (If ever vaginal) How old were you the first time you had vaginal sex? (Drop down with ages)
8. Have you ever used a condom?
   1. Yes
   2. No
9. (If ever used a condom and ever vaginal) How old were you the first time you used a condom for vaginal sex? (Drop down with ages, with check box for “never used)
10. (If ever used a condom and ever anal) How old were you the first time you used a condom for anal sex? (Drop down with ages, with check box for “never used)
11. (If ever used a condom) How would you rate your experience using condoms?
    1. Not very experienced
    2. Somewhat experienced
    3. Very experienced
12. (If ever anal sex) Have you had anal sex in the last year?
    1. Yes
    2. No
13. (If anal sex in the last year) How many times did you have anal sex in the last year?
    1. 12 or more times (at least once a month)
       1. About once a month
       2. About twice a month
       3. About once a week
       4. 2 or more times a week
    2. 1-11 times (less than every month)
       1. Drop down with 1-11
14. (If anal sex 1 time in the last year and ever used condom) Did you use a condom the time that you had anal sex in the past year?
15. Yes (100%)
16. No (0%)
17. (If anal sex 2 times in the last year and ever used condom) Of the 2 times that you had anal sex in the past year, how many times did you use a condom?
18. 0 (0%)
19. 1 (50%)
20. 2 (100%)
21. (If anal sex 3 times in the last year and ever used condom) Of the 3 times you had anal sex in the past year, how many times did you use a condom?
22. 0 (0%)
23. 1 (33%)
24. 2 (67%)
25. 3 (100%)
26. (If anal sex 4-11 or 12 or more times in the last year and ever used condom) Of those times that you had anal sex in the past year, about how often did you use a condom? \
27. Always (100%)
28. Most of the time (75-99%)
29. Sometimes (50-74%)
30. Rarely (1-49%)
31. Never (0%)
32. (If ever vaginal sex) Have you had vaginal sex in the last year?
    1. Yes
    2. No
33. (If vaginal sex in the last year) How many times you have vaginal sex in the last year?
    1. 12 or more times (at least once a month)
       1. About once a month
       2. About twice a month
       3. About once a week
       4. 2 or more times a week
    2. 1-11 times (less than every month)
       1. Drop down 1-11
34. (If vaginal sex 1 time in the last year and ever used condom) Did you use a condom the time that you had vaginal sex in the past year?
35. Yes (100%)
36. No (0%)
37. (If vaginal sex 2 times in the last year and ever used condom) Of the 2 times that you had vaginal sex in the past year, how many times did you use a condom?
38. 0 (0%)
39. 1 (50%)
40. 2 (100%)
41. (If vaginal sex 3 times in the last year and ever used condom) Of the 3 times you had vaginal sex in the past year, how many times did you use a condom?
42. 0 (0%)
43. 1 (33%)
44. 2 (67%)
45. 3 (100%)
46. (If vaginal sex 4-11 or 12 or more times in the last year and ever used condom) Of those times that you had vaginal sex in the past year, about how often did you use a condom? \
47. Always (100%)
48. Most of the time (75-99%)
49. Sometimes (50-74%)
50. Rarely (1-49%)
51. Never (0%)

# Sexual dysfunction

The next few questions will ask about your sexual experience over the past 6 months.

1. (If ever used a condom) Have you used a condom for insertive sex in the last six months?
2. Yes
3. No
4. ~~wIn the past 6 months, how often did you have a desire for sex with your partner? [Source: Adapted from EBAN]~~
   1. ~~Never~~
   2. ~~Rarely~~
   3. ~~Sometimes~~
   4. ~~Often~~
   5. ~~Always~~

First, we are going to ask you about your sexual experience WITHOUT A CONDOM in the past 6 months.

1. When you had insertive sex WITHOUT A CONDOM in the past 6 months: [Source: The International Index of Erectile Function Questionnaire http://www.hiv.va.gov/provider/manual-primary-care/urology-tool2.asp]

| - 1. How do you rate your **confidence** that you could get and keep an erection? | Very low | Low | Moderate | High | Very high |
| --- | --- | --- | --- | --- | --- |
| - 1. When you had erections with sexual stimulation, **how often** were your erections hard enough for penetration? | Almost never/never | A few times (much less than half the time) | Sometimes (about half the time) | Most times (much more than half the time) | Almost always/always |
| - 1. During sexual intercourse, **how often** were you able to maintain your erection after you had penetrated (entered) your partner? | Almost never/never | A few times (much less than half the time) | Sometimes (about half the time) | Most times (much more than half the time) | Almost always/always |
| - 1. During sexual intercourse, **how difficult** was it to maintain your erection to completion of intercourse? | Extremely difficult | Very difficult | Difficult | Slightly difficult | Not difficult |
| - 1. When you attempted sexual intercourse, **how often** was it satisfactory for you? | Almost never/never | A few times (much less than half the time) | Sometimes (about half the time) | Most times (much more than half the time) | Almost always/always |

1. When you had insertive sex WITHOUT A CONDOM in the past 6 months, did you have trouble with any of the following: *Check all that apply.*
   1. Ejaculating too soon
   2. Ejaculating too late
   3. Difficulty ejaculating at all

(If used a condom in the last six months) Now, we are going to ask you about your sexual experience WITH A CONDOM in the past 6 months.

1. (If used a condom in the last six months) When you used a condom for insertive sex in the past 6 months: [Source: The International Index of Erectile Function Questionnaire http://www.hiv.va.gov/provider/manual-primary-care/urology-tool2.asp]

| - 1. How do you rate your **confidence** that you could get and keep an erection? | Very low | Low | Moderate | High | Very high |
| --- | --- | --- | --- | --- | --- |
| - 1. When you had erections with sexual stimulation, **how often** were your erections hard enough for penetration? | Almost never/never | A few times (much less than half the time) | Sometimes (about half the time) | Most times (much more than half the time) | Almost always/always |
| - 1. During sexual intercourse, **how often** were you able to maintain your erection after you had penetrated (entered) your partner? | Almost never/never | A few times (much less than half the time) | Sometimes (about half the time) | Most times (much more than half the time) | Almost always/always |
| - 1. During sexual intercourse, **how difficult** was it to maintain your erection to completion of intercourse? | Extremely difficult | Very difficult | Difficult | Slightly difficult | Not difficult |
| - 1. When you attempted sexual intercourse, **how often** was it satisfactory for you? | Almost never/never | A few times (much less than half the time) | Sometimes (about half the time) | Most times (much more than half the time) | Almost always/always |

1. (If used a condom in the last six months) When you used a condom for insertive sex in the past 6 months, did you have trouble with any of the following: *Check all that apply.*
2. Ejaculating too soon
3. Ejaculating too late
4. Difficulty ejaculating at all

# Condom slippage and breakage

1. (If used a condom in the last six months) During the last six months when you used a condom for [insertive anal (you were the top)/vaginal] sex did any of the following occur? [Source: Element]

|  | Yes | No |
| --- | --- | --- |
| Did you start having sex without a condom and the pull out and put one on? |  |  |
| Did you start having sex with a condom and the pull out and take it off before sex was over? |  |  |
| Did the condom break during sex? |  |  |
| Did the condom slip-off during sex? |  |  |

1. (If ever used a condom) Have you used a condom for insertive sex in the last 30 days?
2. Yes
3. No
4. (If used a condom in the last 30 days and experienced condom slippage and breakage in last six months) During the last 30 days when you used a condom for [insertive anal (you were the top)/vaginal] sex did any of the following occur? [Source: Element]

|  | Yes | No |
| --- | --- | --- |
| Did you start having sex without a condom and the pull out and put one on? |  |  |
| Did you start having sex with a condom and the pull out and take it off before sex was over? |  |  |
| Did the condom break during sex? |  |  |
| Did the condom slip-off during sex? |  |  |

1. (If yes to any condom slippage or breakage in the last six months) You indicated that you have experienced [types of failures from 6mo question] during [insertive anal (you were the top)/vaginal] sex. Approximately how many times has this happened to you in the past 6 months? (When programming, if only 1 or 2 selected in previous question, list as separate questions rather than in a table)

|  | 1 time | 2-4 times | 5-10 times | >= 11 times |
| --- | --- | --- | --- | --- |
| Start sex without a condom, pull out and put one on |  |  |  |  |
| Start sex with a condom, pull out and take it off before finishing sex |  |  |  |  |
| Condom breaking during sex |  |  |  |  |
| Condom slipping off during sex |  |  |  |  |

1. (If yes to any condom slippage or breakage in the last 30 days) You indicated that you have experienced [types of failures from 30 day question] during [insertive anal/vaginal] sex. Approximately how many times has this happened to you in the past 30 days? (When programming, if only 1 or 2 selected in previous question, list as separate questions rather than in a table)

|  | 1 time | 2-4 times | 5-10 times | >= 11 times |
| --- | --- | --- | --- | --- |
| Start sex without a condom, pull out and put one on |  |  |  |  |
| Start sex with a condom, pull out and take it off before finishing sex |  |  |  |  |
| Condom breaking during sex |  |  |  |  |
| Condom slipping off during sex |  |  |  |  |

1. You indicated experiencing [types of failures from previous question] during [insertive anal (you were the top)/vaginal] sex. Did any of the following happen while you were buzzed on alcohol or high on drugs? (When programming, if only 1 or 2 selected in previous question, list as separate questions rather than in a table)

|  | Yes | No |
| --- | --- | --- |
| Start sex without a condom, pull out and put one on |  |  |
| Start sex with a condom, pull out and take it off before finishing sex |  |  |
| Condom breaking during sex |  |  |
| Condom slipping off during sex |  |  |

1. You indicated starting sex without a condom, pulling out and putting one on while having [insertive anal (you were the top)/vaginal] sex. Which of the following are reasons for why this happened? *Check all that apply*.
2. Heat of the moment
3. I / partner need/s to start sex without a condom to get excited
4. I only use condoms to ejaculate
5. Partner asked me to put it on
6. Other, please specify: _______________
7. Of these reasons you selected, which was most important?
8. You indicated starting sex with a condom, pulling out and taking it off before finishing [insertive anal (you were the top)/vaginal] sex. Which of the following are reasons for why this happened? *Check all that apply.*
9. I was losing my erection
10. Partner asked to remove the condom
11. The condom broke
12. The condom was slipping off
13. The condom didn’t fit (it was too small, too large, or the wrong shape)
14. The condom didn’t feel good (it caused irritation on your skin, or you/your partner couldn’t feel anything with it on)
15. Other, please specify: _______________
16. Of these reasons you selected, which was most important?

# Lubricant use

1. In the past 30 days, how often was a lubricant (lube) used during [insertive anal (you were the top) or vaginal] sex, including commercial products, saliva (spit), or any other liquid/gel? [Source: Adapted from Gorbach et al. STI 2012; Element]
2. Always
3. Sometimes
4. Never
5. Don’t know
6. In the past 30 days, when you used a lubricant, which of the following did you use? *Check all that apply.* [Source: Adapted from Gorbach et al. STI 2012; Element]
7. Silicone-based
8. Water-based
9. Stimulating
10. Oil-based (like baby oil or Crisco)
11. Saliva (spit)
12. Lotion
13. Other, please specify: ___________________
14. Don’t know
15. In the past 30 days, when you had sex with a condom, did you use:
    1. Pipe types of lubricant selected with option for none
16. You indicated having used oil based lubricants, saliva, or lotion in the past 30 days, which of the following are reasons you used these types of lubricants: [Source: Element] [show if Q47 = “Oil-based”] Check all that apply.
    1. They are more available than other types of lubricants
    2. They are cheaper than other types of lubricants
    3. I prefer how they feel during sex compared to other types of lubricants
    4. They are what I have at home
    5. Other, please specify: ___________________
17. Please answer the following regarding oil-based lubricants to the best of your knowledge: [Source: Element]

|  | Yes | No | Don’t know |
| --- | --- | --- | --- |
| Using oil-based lubricants during sex with a condom increases the chances that the condom will break |  |  |  |
| Using oil-based lubricants during sex without a condom increases the chances of transmitting HIV and other STIs |  |  |  |

# Therapeutic methods

1. In the past 6 months, which of the following have you used for sexual activity? *Check all that apply.* [Source: PROMIS]
   1. A pill such as Viagra, Cialis, or Levitra
   2. Testosterone
   3. An injection into your penis to get an erection
   4. A vacuum or penis pump to get an erection
   5. A penile implant
2. Do you have a prescription for Viagra, Cialis, or Levitra for sexual activity? [Source: PROMIS] [show if used pill]
   1. Yes
   2. No
3. In the past 6 months, how often have you used an injection into your penis to get an erection? [Source: PROMIS] [show if used injection]
   1. Never
   2. Rarely
   3. Sometimes
   4. Often
   5. Always
4. In the past 6 months, how often have you used a vacuum pump (penis) pump to get an erection? [Source: PROMIS] [show if used pump]
   1. Never
   2. Rarely
   3. Sometimes
   4. Often
   5. Always

# Current condoms used

1. Which of the following types of condoms have you used in the last 6 months? *Check all that apply*. [question mark that you can click if you don’t one what a domed condom is, for example, that will pop up with a picture]
2. Non-Latex
3. Ribbed
4. Studded
5. Textured
6. Colored/Patterned
7. Flavored
8. Domed
9. Thin
10. Magnum
11. Slim fit
12. Other, please specify: ___________________
13. Which type of condom is your favorite? [answer options are those selected as ever used]
14. Where have you typically obtained condoms? *Check all that apply.* [Source: Sibanye]
15. Bought from a pharmacy or drug store
16. Received for a free at a health clinic
17. Received for free at a community-based organization
18. Received for free at a family planning clinic
19. My partner has gotten the condom(s) we used
20. Bought online
21. Other, please specify: ___________________

# Condom fit and feel

1. How much do you agree with the following: (strongly disagree to strongly agree) Condom Perceptions: Negative Condom Attributes subscale [Source: Sanders 2014]
   1. Condoms do not fit me well
   2. Condoms are uncomfortable
   3. Condoms decrease my sensation
   4. Most condoms are not the right shape for me
   5. I do not like the texture of condoms
   6. I do not like the smell of most condoms
2. The last time you used a condom: [Source: Sanders 2014]
3. How did the overall length of this condom fit?
   1. Excellent
   2. Very Good
   3. Just Right
   4. Fair
   5. Poor
4. How did the overall width of this condom fit?
   1. Excellent
   2. Very Good
   3. Just Right
   4. Fair
   5. Poor

# Self-efficacy around condom use

- 1. How confident or sure are you that you could: [Source: Adapted from EBAN]

|  | Not at all confident or sure | Somewhat confident or sure | Very confident/sure |
| --- | --- | --- | --- |
| Put on a condom on your hard penis |  |  |  |
| Unroll a condom down your penis on the first try |  |  |  |
| Start over with a new condom if you placed it on the wrong way |  |  |  |
| Unroll a condom fully to the base of the penis |  |  |  |
| Squeeze air from the tip of a condom |  |  |  |
| Take a condom off without spilling the semen or cum |  |  |  |
| Take a condom off before you lose your hard on |  |  |  |
| Use lubricant with a condom |  |  |  |

# HIV and STI history

This next section will ask some questions about your experiences with HIV testing. We appreciate very much your complete and honest answers to better understand the HIV epidemic in Atlanta.

1. Have you ever had an HIV test? [Source: Involvement, Element]
   1. Yes
   2. No
   3. Don’t know
2. Which of these best describes the most important reason you have not had an HIV test? [Source: NHBS adapted to be lifetime vs past 12 months and reworded; Element] [show if ever HIV test = “No”]
3. Feel at low risk for HIV infection
4. Afraid of finding out that you had HIV
5. Haven’t had the time
6. Haven’t had the opportunity
7. Not sure where to get tested
8. Some other reason, please specify:
9. No particular reason
10. Don’t know
11. In your lifetime, how many HIV tests have you ever had?
12. 1
13. 2
14. 3
15. 4 or more
16. Before today, when did you have your last HIV test? Please choose the month and year. [Source: Element] [show if ever HIV test = “Yes”]
17. Year
18. Month
19. Don’t know
20. Before today, what was the result of your most recent HIV test? [Source: Element] [show if ever HIV test = “Yes”]
21. Negative, you do NOT have HIV
22. Positive, you DO have HIV
23. Did not receive result
24. Don’t know
25. Have you ever tested for HIV for any of these reasons? [Source: D.Katz, Involvement m18/24 limited subset, Element] [show if ever HIV test = “yes”]
26. I had unprotected vaginal sex
27. I had unprotected anal sex
28. I had unprotected oral sex
29. I shared needles or syringes with someone
30. I had sex with someone I knew was HIV positive
31. I had sex with someone and found out afterwards that he/she was HIV positive
32. A sex partner requested it
33. I was worried I might have been exposed to HIV
34. I had symptoms of HIV
35. I had symptoms of an STI other than HIV
36. I get tested on a regular schedule and it was time for me to get tested again
37. I was starting a new relationship
38. My doctor or health care provider recommended I get tested
39. The health department contacted me because one of my sex partners tested positive for HIV
40. Because I got paid to have an HIV test as part of a study
41. I was somewhere testing was being offered for free (Pride, other testing event)
42. Other, please specify: ___________________
43. What was the main reason you got your last HIV test? [Source: Element] [show if HIV test result = “Negative”]
44. Pipe selected options from previous question
45. What was the main reason you got your HIV test, the time when you first tested positive for HIV? [Source: Element] [show if HIV test result = “Positive”]
46. Pipe selection options from why tested

A sexually transmitted infection (STI) is an infection transmitted through sexual activity such as syphilis, gonorrhea, chlamydia, herpes, or genital warts.

1. Have you ever been told you have an STI? [Source: Involvement, Element]
2. Yes
3. No
4. Don’t know
5. In the past 12 months, did any of these happen to you, as part of testing or examination for an STI that was not HIV? [Source: Element]
6. Urinated (peed) in a cup
7. Used a swab on the opening of my penis (meatus), or had it swabbed by someone else
8. Used a swab on my rectum, or had it swabbed by someone else
9. Had my throat swabbed
10. Had my blood drawn
11. During the past 12 months, has a doctor, nurse, or other health care worker told you that you had either of the following? *Check all that apply.* [Source: Element]
12. Syphilis
13. Gonorrhea (clap or drip)
14. Chlamydia
15. Herpes
16. Genital warts
17. Other, please specify
18. You said that you were told you had [answer from previous question]. Were you then treated for these STIs? [Source: Element] [show if STI result = “Syphilis” or “Gonorrhea”]
19. Yes
20. No

# Partner numbers and names in last 30 days

1. In the last 6 months, with how many people did you have vaginal or anal sex?
2. In the last 30 days, with how many people did you have vaginal or anal sex?
3. The next section of this survey will ask about your recent sexual partner(s). To do this, we will first ask you to provide a first name for each of your recent partner(s). We will use the name of each partner to fill in future survey questions, like: “Does (name) have genital piercings?”

• Please use your partner’s first name only (we don’t need or want the last name).

• If you do not know or don’t want to use your partner’s name, you can use a nickname. Use a name you will remember. We will be using this name throughout your study participation (this and future surveys) to make it easier to understand study questions

1. List 3 most recent partners
2. You mentioned that you are [married/in a registered domestic partnership or civil union]. Is [any of these] your married partner? If yes, check which: [show if marital status = “Legally married” or “Registered domestic partnership or civil union”]
3. List of partners

# Partner-specific questions (Element)

1. Is [partner name] male or female?
   1. Male
   2. Female
2. What is the current age of [partner name]? (Drop-down: Don’t know, 18-80, older than 80)
3. Does [partner name] consider himself/herself (depending on partner gender) to be Hispanic or Latino?
   1. Yes
   2. No
   3. Don’t know
4. What racial group does [partner name] consider himself/herself (depending on partner gender) to be in?
   1. White
   2. Black or African American
   3. American Indian or Alaska Native
   4. Asian
   5. Native Hawaiian or other Pacific Islander
   6. Multi-racial
   7. Other
   8. Prefer not to answer
5. A main partner is someone that you feel committed to above all others -- this is someone that you might call your boyfriend/girlfriend, significant other, or life partner.
   Is [partner name] a main partner or a casual partner?
   1. Main partner
   2. Casual partner
6. In the last 30 days, how many times have you had [vaginal/anal] with [partner name]:
7. 0
8. 1
9. 2
10. 3
11. 4 or more times
12. About how often did you have [vaginal, anal] sex with [partner name] per week? [show if sex in last 30 days = “4 or more times”]
13. About once a week
14. 2-4 times a week
15. 5-6 times a week
16. Every day
17. Did you use a condom the time you had [vaginal/anal] sex with [partner name]? [show if sex with partner = “1”]
18. Yes (100%)
19. No (0%)
20. Of the 2 times you had [vaginal/anal] sex with [partner name], how many times did you use a condom? [show if sex with partner = “2”]
21. 0 (0%)
22. 1 (50%)
23. 2 (100%)
24. Of the 3 times you had [vaginal/anal] sex with [partner name], how many times did you use a condom? [show if sex with partner = “3”]
25. 0 (0%)
26. 1 (33%)
27. 2 (67%)
28. 3 (100%)
29. About how often did you use a condom during the times you had [vaginal, anal] sex with [partner name]? [show if sex with partner = “4 or more”]
30. Always (100%)
31. Most of the time (75-99%)
32. Sometimes (50-74%)
33. Rarely (1-49%)
34. Never (0%)
35. Imagine you have completed the study.
    1. If a condom from the brand you currently prefer was available and you were about to have sex with **[partner name]**, would you use the condom?
       1. Yes
       2. No
    2. If a condom from the brand you currently prefer was available and you were about to have sex with **a new partner**, would you use the condom?
       1. Yes
       2. No
36. Are you currently using a form of birth control other than condoms with [partner name]? [show if female partner]
37. Yes
38. No
39. What kind of birth control are you using with [partner name]? [show if birth control = “yes”]
40. Depo-Povera or injectable
41. Female hormones in the form of pill, cream, or patch
42. Intrauterine device (IUD)
43. Don’t know
44. Do you plan to have sex with [partner name] again?
45. Yes
46. No
47. Does [partner name] have [vaginal/anal] piercings?
48. Yes
49. No
50. Do you know [partner name]’s HIV status?
51. Yes, HIV-positive
52. Yes, HIV-negative
53. Don’t know
54. Is [partner name] currently in treatment? [show if HIV status is positive]
55. Yes
56. No
57. With [partner name], who initiates condom use? [show if condom use with partner ≠ “Never”]
58. Me
59. [partner name]
60. Both of us
61. With [partner name], who brings condoms? [show if condom use with partner ≠ “Never”]
62. Me
63. [partner name]
64. Both of us
65. How willing would you be to do the following: [Source: Adapted from EBAN]

|  | Very unlikely | Unlikely | Somewhat likely | Likely | Very likely |
| --- | --- | --- | --- | --- | --- |
| Discuss condom use with [partner name] |  |  |  |  |  |
| Insist on condom use if [partner name] does not want to use one |  |  |  |  |  |
| Stop and look for condoms when you’re sexually aroused |  |  |  |  |  |
| Insist on condom use every time even when you are under the influence of alcohol or drugs |  |  |  |  |  |
| Insist on condom use every time even when your partner is under the influence of alcohol or drugs |  |  |  |  |  |
| Put a condom on without spoiling the mood |  |  |  |  |  |
| [MSW Only] Insist on condom use every time even if [partner name] uses another method to prevent pregnancy |  |  |  |  |  |
| Say NO to having sex with [partner name] if he/she was pressuring you to have sex |  |  |  |  |  |
| Say NO to having sex with [partner name] if he/she refused to use a condom |  |  |  |  |  |

1. How willing would [partner name] be to do the following:

|  | Very unlikely | Unlikely | Somewhat likely | Likely | Very likely |
| --- | --- | --- | --- | --- | --- |
| Discuss condom use with you |  |  |  |  |  |
| Insist on condom use if you do not want to use one |  |  |  |  |  |
| Stop and look for condoms when [he/she] is sexually aroused |  |  |  |  |  |
| Insist on condom use every time even when [he/she] is under the influence of alcohol or drugs |  |  |  |  |  |
| Insist on condom use every time even when you are under the influence of alcohol or drugs |  |  |  |  |  |
| Put a condom on without spoiling the mood |  |  |  |  |  |
| [MSW Only] Insist on condom use every time even if she uses another method to prevent pregnancy |  |  |  |  |  |
| Say NO to having sex with you if you were pressuring him/her to have sex |  |  |  |  |  |
| Say NO to having sex with you, if you refused to use a condom |  |  |  |  |  |

# Physical Sexual Pleasure

Think about the last time you had sex. For the next set of questions, we are going to ask you to rate a number of experience about the last time you had sex. Please move the slider to show how much you agree with each. (EMSEXpleasure)

1. Which of these partners did you most recently have sex with?
2. List of partners
3. Did you use a condom?
4. Yes
5. No
6. Did you ejaculate (cum)?
7. Yes, in the condom
8. Yes, not in the condom
9. No
10. (If ejaculated in the condom) The orgasm was outstanding.
11. Strongly disagree – Strongly agree (slider)
12. (If ejaculated in the condom) The timing of my ejaculation (cum) was just right.
13. Strongly disagree – Strongly agree (slider)
14. The physical sensation on my penis was outstanding.
15. Strongly disagree – Strongly agree (slider)
16. This sex was very pleasurable.
17. Strongly disagree – Strongly agree (slider)
18. I was able to maintain my erection throughout the sex act.
19. Strongly disagree – Strongly agree (slider)
20. The firmness of my erection was ideal during sex.
21. Strongly disagree – Strongly agree (slider)
22. My penis was comfortable during sex (for example, not pinched).
23. Strongly disagree – Strongly agree (slider)
24. I was highly physically aroused during sex.
25. Strongly disagree – Strongly agree (slider)

Question sources:

**Involvement:** Sullivan PS, Rosenberg ES, Sanchez TH, Kelley CF, Luisi N, Cooper HL, et al. Explaining racial disparities in HIV incidence in black and white men who have sex with men in Atlanta, GA: a prospective observational cohort study. Annals of epidemiology. 2015;25(6):445-54.

**Element:** Serota DP, Rosenberg ES, Lockard AM, Rolle C-PM, Luisi N, Cutro S, et al. Beyond the Biomedical: Preexposure Prophylaxis Failures in a Cohort of Young Black Men Who Have Sex With Men in Atlanta, Georgia. Clinical Infectious Diseases. 2018:ciy297-ciy.

**MMP:** Do AN, Rosenberg ES, Sullivan PS, Beer L, Strine TW, Schulden JD, et al. Excess burden of depression among HIV-infected persons receiving medical care in the United States: data from the medical monitoring project and the behavioral risk factor surveillance system. 2014;9(3):e92842.

**NHBS:** Centers for Disease C, Prevention. Prevalence and awareness of HIV infection among men who have sex with men --- 21 cities, United States, 2008. MMWR Morbidity and mortality weekly report. 2010;59(37):1201-7.

**Sibanye:** McNaghten A, Kearns R, Siegler AJ, Phaswana-Mafuya N, Bekker LG, Stephenson R, et al. Sibanye Methods for Prevention Packages Program Project Protocol: Pilot Study of HIV Prevention Interventions for Men Who Have Sex With Men in South Africa. JMIR Res Protoc. 2014;3(4):e55

**EBAN:** El-Bassel N, Jemmott JB, Landis JR, Pequegnat W, Wingood GM, Wyatt GE, et al. National Institute of Mental Health Multisite Eban HIV/STD Prevention Intervention for African American HIV Serodiscordant Couples: a cluster randomized trial. Archives of internal medicine. 2010;170(17):1594-601.

**PROMIS:** Broderick JE, DeWitt EM, Rothrock N, Crane PK, Forrest CB. Advances in Patient-Reported Outcomes: The NIH PROMIS((R)) Measures. EGEMS (Washington, DC). 2013;1(1):1015.

**EMSEXpleasure:** Siegler AJ, Boos E, Rosenberg ES, Cecil MP, Sullivan PS. Validation of an Event-Level, Male Sexual Pleasure Scale (EMSEXpleasure) Among Condom-Using Men in the U.S. Arch Sex Behav. 2018.
